# Advances in precision oncology using patient-derived organoids and functional biomaterials

**DOI:** 10.3389/fcell.2025.1670328

**Published:** 2025-09-30

**Authors:** Hina Singh, Ivan Mijakovic, Priyanka Singh

**Affiliations:** ^1^ Division of Biomedical Sciences, School of Medicine, University of California, Riverside, Riverside, CA, United States; ^2^ The Novo Nordisk Foundation, Center for Biosustainability, Technical University of Denmark, Kogens Lyngby, Denmark; ^3^ Systems and Synthetic Biology Division, Department of Biology and Biological Engineering, Chalmers University of Technology, Gothenburg, Sweden

**Keywords:** patient-derived organoids, precision oncology, biomaterials, organ-on-chip, 3D culture, immuno-oncology, microfluidic platforms, translational modeling

## Abstract

Despite major advances in oncology, cancer therapy continues to face persistent challenges due to intratumoral heterogeneity, drug resistance, and the poor clinical translation of experimental therapeutics. Conventional preclinical models such as 2D cultures and animal systems often fail to accurately recapitulate the tumor microenvironment immune contexture, and patient-specific variability limiting their predictive power. While nanomedicine and advanced drug delivery platforms offer promising solutions, their translational success is hindered by insufficient integration with physiologically relevant tumor models. In this review, we critically examine how patient-derived organoids derived from patient tumors serve as next-generation platforms for modeling cancer heterogeneity, therapeutic response, and biomarker discovery. We further explore how the integration of PDOs with functional biomaterials, extracellular matrix mimetics, and organ-on-chip systems enables dynamic co-culture environments that capture tumor–stroma–immune interactions with high fidelity. By linking the biological underpinnings of resistance, such as genetic mutations, altered signaling, metabolic rewiring, and immune evasion, with smart biomaterial design and drug screening workflows, we propose a unified roadmap for precision oncology. Additionally, we highlight the emergence of PDO biobanks, co-culture innovations, and high-throughput phenotypic screening as essential tools for improving clinical translation. This interdisciplinary synthesis underscores the transformative potential of PDO-based platforms in accelerating personalized cancer therapy.

## Graphical Abstract

**Figure:**
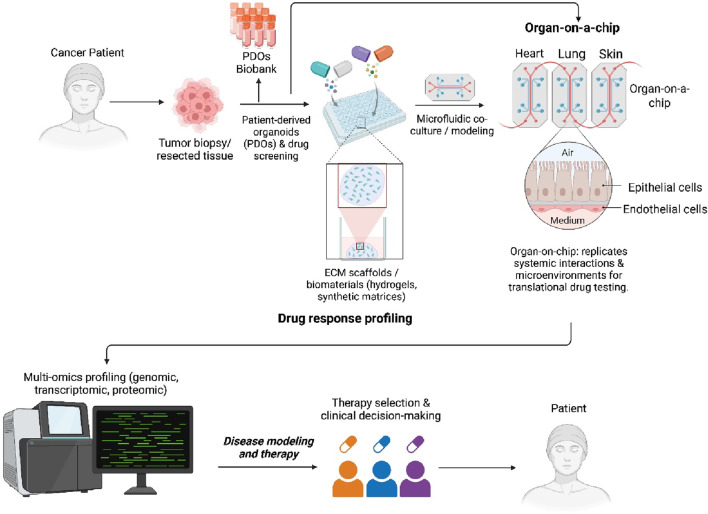
PDOs enable drug screening, multi-omics profiling, and integration with biomaterials and organ-on-chip platforms to guide personalized therapy and advance precision oncology.

## 1 Introduction

As of 2025, cancer remains one of the leading causes of death worldwide. According to the World Health Organization’s International Agency for Research on Cancer (IARC), an estimated 20 million new cancer cases and 9.7 million cancer-related deaths occurred globally in 2022, with lung, breast, and colorectal cancers being the most prevalent types (Siegel et al., 2025; Bray et al., 2024). It is estimated that about one in five men and women will develop cancer at some point in their lives, while roughly one in nine men and one in 12 women will die from it. For males, there were 10.3 million new cases and 5.4 million deaths; for females, there were 9.7 million new cases and 4.3 million deaths (Bray et al., 2024). The most commonly diagnosed cancers were lung (2.5 million cases; Taverna et al., 2024), breast (2.3 million), and colorectal (1.9 million). In the United States alone, an estimated 2,041,910 new cancer cases and 618,120 cancer deaths are projected for 2025 (Siegel et al., 2025). Despite increasing survival from early detection and advanced therapies, the overall burden continues to rise, highlighting the need for more effective personalized approaches.

Surgery remains one of the most used treatment options, particularly for early-stage cancers where complete removal of the tumor is possible (Anusha et al., 2025). Minimally invasive techniques such as laparoscopic and robotic-assisted surgery have improved patient recovery and reduced complications. Radiation therapy is another widely used treatment, involving the use of high-energy radiation to destroy cancer cells (Tiruye et al., 2023; Yamoah et al., 2015). Advanced techniques, such as intensity-modulated radiation therapy (IMRT) and proton beam therapy, have enhanced the precision of radiation delivery (Vaios and Wo, 2020). Chemotherapy continues to be a key component of cancer treatment, particularly for metastatic cancers where surgical options are limited. It is frequently employed as a primary therapy for advanced cancers, a neoadjuvant treatment to shrink tumors before surgery, and an adjuvant therapy to eliminate residual cancer cells post-surgery (Wei et al., 2021). Traditional chemotherapy drugs primarily target rapidly dividing cells; however, their lack of specificity often leads to off-target toxicity, resulting in side effects such as anemia, infections, and gastrointestinal complications that significantly impact patients’ quality of life (Li B. et al., 2022; Ferro et al., 2023). To address these challenges, newer formulations have focused on improving specificity and reducing toxicity through strategies such as nanomaterials formulations, liposomal drug delivery, and combination with other kind of targeted therapies (Singh et al., 2025).

Targeted cancer therapy (TCT) has become increasingly important, with drugs designed to attack specific molecules involved in cancer growth (Yin et al., 2020; Kaidar-Person et al., 2019). Targeted therapies offer high specificity, reducing neutropenia, off-target toxicity, and multi-drug resistance while enabling higher cytotoxic at the target. In the past few years, many drugs in combination with different biomaterials, proteins, nanoparticles, *etc.*, have been developed based on the principle of active targeting. For instance, Mirvetuximab soravtansine, approved in late 2022, targets folate receptor alpha-positive ovarian cancer resistant to platinum-based chemotherapy (Moore et al., 2023; Matulonis et al., 2023). Enhertu (trastuzumab deruxtecan), approved in 2024, is an antibody-drug conjugate for HER2-positive solid tumors, combining trastuzumab with a topoisomerase inhibitor for targeted delivery of cytotoxic agents (Li B. T. et al., 2022; Nakada et al., 2019). Lifileucel (Amtagvi), approved in February 2024, is the first FDA-approved tumor-infiltrating lymphocyte (TIL) therapy for metastatic melanoma (Sarnaik et al., 2021; Chesney et al., 2022). Zanidatamab (Ziihera), approved in November 2024, is a bispecific antibody targeting two HER2 receptor sites for treating HER2-positive biliary tract cancer (Harding et al., 2023; Meric-Bernstam et al., 2022). Inavolisib (Itovebi), approved in October 2024, is a PI3K alpha inhibitor used for PIK3CA-mutant breast cancer, a mutation commonly found in several cancers (Blair, 2025). Sacituzumab govitecan (Trodelvy), initially approved in 2020 for metastatic triple-negative breast cancer, has since expanded to treat hormone receptor-positive, HER2-negative breast cancer and metastatic urothelial cancer by delivering a topoisomerase inhibitor directly to tumor cells (Bardia et al., 2021; Goldenberg et al., 2015).

In addition to TCT, immunotherapy has also become a key strategy in cancer treatment by enabling the body’s immune system to recognize and eliminate cancer cells. Immune checkpoint inhibitors, such as PD-1 and PD-L1 inhibitors, have shown remarkable efficacy in melanoma, lung cancer, and other solid tumors (Rapoport et al., 2021; Bukamur et al., 2020). In December 2024, the FDA approved Imfinzi (durvalumab) for limited-stage small cell lung cancer, enhancing the immune response by blocking the PD-L1 pathway (Durvalumab Imfinzi, 2023; Patel et al., 2020; Westin et al., 2024). CAR-T cell therapy, which involves modifying a patient’s T cells to target cancer, has shown promising results in hematological cancers like leukemia and lymphoma (Dabas and Danda, 2023). Hormone therapy remains a standard treatment for hormone-dependent cancers such as breast and prostate cancer, with drugs targeting estrogen, androgen, and other hormone pathways. In January 2025, the FDA approved datopotamab deruxtecan-dlnk (Datroway) for hormone receptor-positive, HER2-negative breast cancer, delivering chemotherapy directly to cancer cells while sparing healthy tissue (Bardia et al., 2024). These recent advancements underscore the growing precision and efficacy of immunotherapy, CAR-T cell therapy, and hormone-based treatments in improving cancer outcomes.

Similarly, many nanomedicines in combination with radiation therapies have played a critical role in cancer treatment, such as cancer cell membrane-coated nanoparticles that enhance targeted drug delivery and phototherapy efficacy by improving tumor targeting and reducing immune clearance (Singh et al., 2025; Bhatia et al., 2021). Another example is Photodynamic therapy (PDT), which involves light-activated drugs, has been enhanced by nanomedicines to boost the immune response, improving anti-tumor effects in combination therapies (Sun et al., 2022). Gene therapy has also advanced, with strategies targeting specific genetic mutations driving cancer progression, such as using CRISPR-based editing to correct mutations in hematologic cancers. Stem cell transplants have become more accessible due to the use of reduced-intensity conditioning regimens, which improve outcomes in older patients with leukemia and lymphoma (Bhatt et al., 2018). Other approaches for cancer treatment are Autogene Cevumeran, a personalized mRNA vaccine, which has shown promise in clinical trials for pancreatic cancer (Rojas et al., 2023). Oncolytic viruses (OVs), such as RP2, an engineered herpes simplex virus currently in clinical trials for melanoma and other solid tumors, have demonstrated encouraging results in patient survival (Kalafati et al., 2023). These advancements reflect the dynamic and evolving landscape of cancer therapy, offering more targeted and effective treatments.

Despite advancements in cancer diagnosis and treatment, the complexity of tumor biology, including tumor heterogeneity and the TME, continues to hinder the development of effective, personalized therapies (Brouwer et al., 2024; Faubert et al., 2020). Traditional preclinical models, including 2D cell cultures, spheroids, organoids, animal models, and 3D bioprinting, have long been utilized to study cancer biology and evaluate therapies (Liu et al., 2023). However, these models often fail to replicate the complex *in vivo* environment, cellular diversity, and genetic dynamics of human tumors (Vitale et al., 2022). For instance, organoids and spheroids, while valuable models, are static systems that often face challenges with reproducibility. Tissue engineering struggles to achieve precise cell placement, and 3D bioprinting methods fail to fully replicate key *in vivo* features such as fluid dynamics and biomimetic tissue organization (Leverant et al., 2024). Additionally, experimental animals differ inherently from humans, limiting their ability to accurately predict human responses (Gois et al., 2020). As a result, their limited predictive accuracy contributes to the high failure rate of cancer drugs in clinical trials, with over 90% failing to translate from preclinical studies to successful treatments. This highlights the pressing need for more biomimetic models that can accurately simulate human tumor environments and aid in the development of highly precise, personalized medications.

Recent reviews and translational studies have underscored the growing clinical relevance of PDOs. They have highlighted their value in modeling tumor heterogeneity and drug responses, their application in biomarker discovery and personalized therapy, and provided translational evidence by demonstrating how PDOs can guide therapeutic decisions. While these works collectively establish the promise of PDOs, few have critically evaluated how biomaterials, microfluidic platforms, and dynamic culture systems can synergize with PDOs to enhance clinical translation (Yang and Yu, 2023; Tong et al., 2024). In this review, we have explored the evolution of PDOs, highlighting diverse approaches such as organ-on-chip and vessel-on-chip technologies, ongoing clinical trials, the integration of emerging technologies, and the current biomaterials employed in their development, along with the challenges that continue to hinder their translational potential.

## 2 PDOs: a paradigm shift

Patient-derived xenografts (PDX) and PDO models have emerged as powerful and complementary tools in cancer research, each offering distinct advantages and addressing specific limitations of traditional models (Kumari et al., 2022). Unlike traditional models, PDOs and PDX retain patient-specific mutations, epigenetic modifications, and drug response profiles over multiple passages (Fashemi et al., 2023). PDX models are established by implanting patient-derived tumor tissue into immunodeficient mice, which allows the tumor to grow in an *in vivo* environment that preserves the original TME, including stromal and vascular components. This makes PDX models particularly useful for studying tumor-stroma interactions, metastasis, and systemic drug responses. In contrast, PDOs are miniature, self-organizing structures cultured from patient tumor tissues, which replicate the genetic, proteomic, and morphological characteristics of the original tumor (Bengtsson et al., 2021; Cruz-Acuna and Garcia, 2019). PDO development involves isolating tumor cells from biopsies or resected tissues, enzymatically digesting them into single cells or clusters, and embedding them in extracellular matrix (ECM)-based scaffolds, such as Matrigel™ (Cruz-Acuna and Garcia, 2019). The culture medium is supplemented with essential growth factors, including Wnt, R-spondin, and epidermal growth factor (EGF), to support cell proliferation and differentiation (Cioce et al., 2023). Within a few days to weeks, these cells self-organize into 3D structures that recapitulate the histology and functionality of the parent tumor.

**FIGURE 1:**
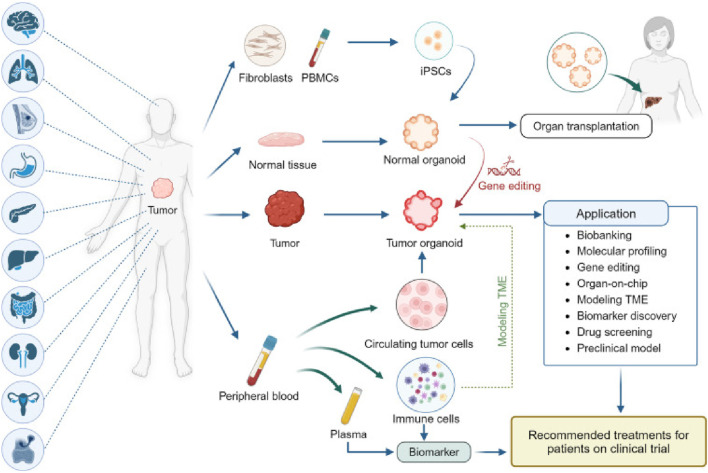
Establishment and application of PDO biobanks: The currently established biobanks of PDOs, sourced from various cancer types. These biobanks collect tumor tissue, paired normal tissue, and blood, with some also having patient-derived iPSCs that are reprogrammed from fibroblasts or peripheral blood mononuclear cells (PBMC)s. Normal organoids derived from iPSCs or normal tissue offer potential in organ transplantation and can be transformed into tumor organoids through gene editing (Tong et al., 2024).

One of the key advantages of PDOs is their ability to retain patient-specific genomic and phenotypic characteristics over multiple passages, unlike 2D cultures, which often undergo clonal drift and loss of critical mutations (Taverna et al., 2024). PDOs can be established across diverse cancer types such as colorectal, breast, lung, pancreatic, and ovarian with relatively high success rates. Their compatibility with high-throughput drug screening and multi-omics profiling has made them an efficient platform for identifying novel therapeutic targets and guiding personalized treatment strategies. In contrast to tumor spheroids and other static 3D systems, PDOs better replicate tumor-specific architecture, cellular heterogeneity, and microenvironmental gradients (e.g., oxygen, nutrients), enhancing their translational relevance (Sisakht et al., 2025; Joshi et al., 2024). Advances in co-culture techniques now allow PDOs to be integrated with stromal cells, fibroblasts, and immune components, enabling the study of tumor–stroma and tumor–immune interactions in a patient-specific context. Compared to PDXs, which maintain stromal architecture *in vivo* but are costly, slow to establish, and unsuitable for high-throughput use, PDOs offer a faster, more scalable, and ethically favorable alternative. Emerging PDO-PDX matched models further strengthen the translational pipeline by enabling *in vitro* drug screening with *in vivo* validation. While PDX models remain essential for systemic response studies, the rise of large PDO biobanks has accelerated early-stage therapeutic testing and precision oncology development (Figure 1). Yet, current PDO biobanks face critical limitations. The absence of harmonized protocols for tissue processing, culture maintenance, and data annotation across centers hinders comparability and poses challenges for collaborative, large-scale clinical translation (Kumari et al., 2022).

Moreover, creating PDOs from adult stem cells (ASCs) within PDX tissue has emerged as an innovative strategy for generating matched *in vitro/in vivo* models. These models retain the genomic, histological, and pharmacological profiles of the original tumor, allowing researchers to conduct higher-throughput *in vitro* screens and validate findings *in vivo* using the corresponding PDX model. Compared to PDXs, which require the implantation of tumors into immunodeficient mice, PDOs provide faster generation times, reduced ethical concerns, and scalability for high-throughput applications. Thus, each model used to study cancer therapies offers distinct advantages and comes with certain limitations. Table 1 summarizes the key properties and differences among 2D cell cultures, 3D spheroid models, PDXs, PDOs, and other models commonly employed in cancer research. It highlights their relative strengths and limitations in replicating tumor heterogeneity, mimicking the TME, scalability, and suitability for drug screening and personalized medicine. Despite the advantages, the establishment of PDOs remains highly dependent on tumor sample quality. Low-cellularity biopsies, necrotic tissue, or samples with excessive stromal content frequently compromise PDO viability, limiting their expansion and downstream applications in drug screening and personalized medicine. Nevertheless, considerable inter-patient variability in PDO growth efficiency and phenotype remains a major barrier, often complicating therapeutic predictions and limiting cross-study comparability. Furthermore, differences in ECM scaffolds, growth factor supplementation, and media formulations between laboratories highlight the lack of standardized culture conditions, reducing reproducibility and clinical consistency.

**TABLE 1:** Comparison of PDOs with traditional preclinical cancer models.

Feature	2D cell cultures	3D spheroids	PDX	PDO
Tumor Fidelity	Low, Loss of architecture, genetic drift	Moderate, improves structure, lacks heterogeneity	High, maintains tumor heterogeneity, includes stroma	High, Preserves genetic, proteomic, and architectural features
Scalability	High, Simple expansion	Moderate, more scalable than PDXs	Low, Costly, slow to expand	High, Suitable for high-throughput formats
Cost	Low, Minimal reagents	Moderate, Needs ECM, bioreactors	High, Requires animals, surgical teams	Moderate, High media cost but no animals
Ethical Concerns	Minimal, no animal use	Low, *in vitro* system	High, Animal welfare and human tissue handling	Low, Derived from patient tissue, no animals
Drug Response Accuracy	Poor, fails to mimic *in vivo*	Moderate, Some prediction capacity	High, reflects *in vivo* drug response	High, Correlates with clinical outcomes
Immune System Modeling	Absent	Limited, Static immune co-cultures possible	Poor to Moderate, Humanized mouse models emerging	Moderate to High, Co-culture with autologous PBMCs or TILs possible
Reproducibility	High, Protocols standardized	Moderate, Variability in ECM and cell lines	Low, Patient and animal variability	Moderate to High, still affected by patient tumor quality and heterogeneity
Clinical Relevance	Low	Moderate, Useful for mechanism studies	High, Predictive of patient outcome	High, enables personalized therapy and biomarker testing
Safety	High, No patient risk	Moderate, No systemic modeling	Moderate, Zoonosis and immunocompromised animal risks	High, Safe if sourced and cultured properly
Mutation and Genetic Drift	High, Rapid loss over time	Moderate, Some preservation	Low, Stable mutation profile	Low, maintains heterogeneity over passages
Time to Establishment	Fast, 1–3 days	Moderate, 1–2 weeks	Slow, 6–12 weeks	Moderate,1–3 weeks
Use in Drug Screening	High, HTS-compatible	Moderate-Medium-throughput assays	Low, not HTS feasible	High, Used in HTS, functional testing, multi-omics analysis

## 3 PDOs in cancer research and personalized therapy

In this section, we explore how PDOs are being utilized to address three critical aspects of cancer research and care: (i) drug sensitivity and resistance profiling, (ii) modeling tumor evolution and genetic heterogeneity, and (iii) advancing immuno-oncology applications. Together, these dimensions underscore the increasing clinical and translational significance of PDOs in the development of personalized and effective cancer therapies.

### 3.1 Drug sensitivity and resistance testing

**FIGURE 2:**
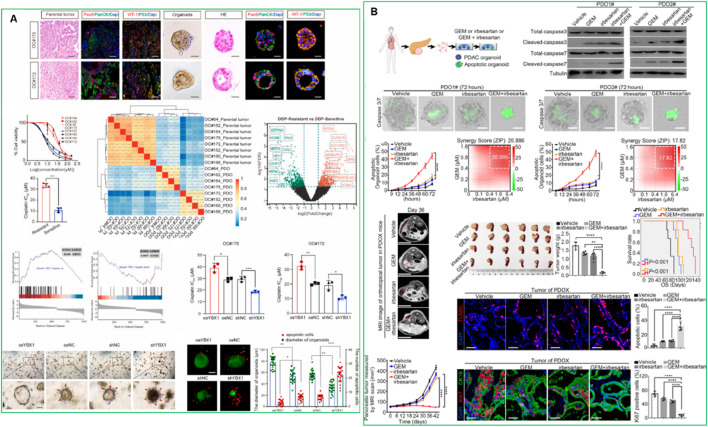
**(A)** High expression of YBX1 reduces sensitivity to platinum drugs in ovarian cancer organoids (Meng et al., 2024). This figure was adapted from **(B)**. Irbesartan efficiently overcomes GEM resistance of PDAC in PDO, PDX, and GEM-resistant BxPC-3 models *in vitro* and *in vivo* (Zhou et al., 2023a). Adapted with permission.

PDOs are particularly valuable for identifying optimal drug combinations and overcoming acquired resistance. By exposing PDOs to a range of chemotherapeutic and targeted agents, researchers can evaluate the efficacy and toxicity of various treatments in a patient-specific context. It significantly reduces the time required to identify optimal therapies, a particularly critical factor for patients with aggressive or rapidly progressing cancers (Luo et al., 2023). Recent studies have established PDO-based drug screening platforms capable of predicting patient response with high accuracy in different cancer models. Georgios et al., 2018. established a living biobank of PDOs from metastatic, heavily pre-treated colorectal and gastroesophageal cancer patients enrolled in phase I/II trials. The PDOs closely mirrored the original tumors in both phenotype and genotype. Drug screening results aligned with molecular profiling, and *ex vivo* PDO responses, as well as PDO-derived xenograft models, correlated with patient outcomes (Vlachogiannis et al., 2018). Ooft et al. (2019) demonstrated that PDOs from metastatic colorectal cancer can predict patient response to irinotecan-based chemotherapy with over 80% accuracy, helping identify non-responders and avoid ineffective treatment (Ooft et al., 2019). These findings support the potential of PDOs to guide personalized treatment strategies and predict clinical responses. Different studies showed that PDOs derived from colorectal cancer patients have successfully predicted sensitivity to chemotherapy agents like 5-fluorouracil (5-FU) and irinotecan (Smabers et al., 2024). This predictive capability has paved the way for high-throughput screening (HTS) of drug libraries, facilitating high-throughput drug screening and enabling the identification of personalized treatment strategies (Smabers et al., 2024). Meng et al. (2024) showed that in ovarian cancer, resistance to platinum-based chemotherapy (e.g., cisplatin) remains a major challenge (Meng et al., 2024). The authors identified the YBX1/m5C-CHD3/HR repair signaling axis as a key mechanism for platinum resistance in ovarian cancer. Inhibiting YBX1 increased sensitivity to platinum-based chemotherapy, highlighting YBX1 as a potential target for overcoming platinum resistance in ovarian cancer using PDO models (Figure 2A). (Meng et al., 2024) Another example by Zhou et al., who demonstrated that irbesartan could enhance chemotherapy efficacy in PDAC patients with high c-Jun expression by inhibiting the Hippo/YAP1/c-Jun/stemness/iron metabolism axis using PDO models (Zhou et al., 2023a). This finding led to the initiation of a phase II clinical trial to evaluate the safety and efficacy of irbesartan combined with a standard gemcitabine/nab-paclitaxel regimen in advanced stage III/IV PDAC (Figure 2B) (Zhou et al., 2023a).

Subsequent research revealed that combining trastuzumab and pertuzumab in HER2-positive breast cancer PDOs yielded enhanced efficacy (Hurvitz et al., 2023). This finding aligns with clinical evidence from the DESTINY-Breast03 trial, which showed a significant improvement in progression-free survival with trastuzumab deruxtecan *versus* trastuzumab emtansine in patients with HER2-positive metastatic breast cancer (Hurvitz et al., 2023). The aim of DESTINY-Breast03 was to compare the efficacy and safety of trastuzumab deruxtecan with trastuzumab emtansine, ultimately reaffirming trastuzumab deruxtecan as the preferred second-line treatment due to its longer progression-free survival and manageable safety profile (Hurvitz et al., 2023). Another study showed that PDOs derived from gastric cancer tissues accurately predicted patient responses to chemotherapy. In one case, the PDO correctly identified sensitivity to capecitabine and oxaliplatin, while in another, it predicted insensitivity to S-1 chemotherapy (Zu et al., 2023). Six of eight cases showed consistency between PDO drug sensitivity results and clinical outcomes (Zu et al., 2023) A separate study further highlighted that PDO-based drug testing in CRC can predict patient responses with 75% sensitivity and specificity. PDOs were established from surgical and core needle biopsies (61.5% success rate) and tested against 25 FDA-approved drugs (Cartry et al., 2023). The authors identified sensitive drugs in 92% of cases, with a strong correlation to clinical outcomes and 94% concordance with the tumor’s genomic profile (Cartry et al., 2023). These examples underscore the growing value of PDO-based drug screening in predicting patient responses, identifying mechanisms of drug resistance, and facilitating the development of TCTs. Despite these encouraging results, variation in inter-patient response rates, lack of harmonized culture protocols, and limited multi-center validation studies remain major challenges for translation.

### 3.2 Tumor evolution and genetic heterogeneity

Tumor heterogeneity, driven by genetic mutations and epigenetic changes, plays a key role in disease progression and treatment resistance. Tumors comprise diverse cellular subpopulations, which complicate therapy by allowing the emergence of resistant clones. PDOs to some extent capture this intra-tumoral heterogeneity, retaining the genetic, epigenetic, and cellular diversity of the original tumor, including cancer stem cells, immune cells, and stromal components. This makes them ideal for studying tumor evolution and the development of drug resistance. Multi-omics profiling (including genomics, transcriptomics, and metabolomics) using PDOs has identified key mutations associated with resistance. So far, scientists have studied the co-culture of PDOs with immune cells and fibroblasts, which enhances the ability to study tumor-stroma and immune interactions. Hypoxia and nutrient availability have also been modeled in PDOs to study how these factors drive tumor evolution and therapy resistance. For instance, Liu et al. (2024) recently demonstrated that in colorectal cancer (CRC), a CRISPR-Cas9 genome-wide screening approach using a spleen-injected liver metastasis mouse model identified ANKRD42 as a key regulator of CRC liver metastasis in PDO models (Liu S. et al., 2024). Elevated ANKRD42 expression was confirmed in metastases from the TCGA database and clinical cohorts. Depleting ANKRD42 in CRC-derived PDOs downregulated genes linked to epithelial-mesenchymal transition (EMT), such as CDH2 and SNAI2, thereby inhibiting tumor migration, invasion, and liver metastasis (Liu S. et al., 2024). Similarly, recent studies have extended functional precision oncology approaches to CRC using quantitative phosphoproteomic analysis of PDOs. This strategy involves perturbing primary tumor cells with kinase inhibitors and measuring proteome activity landscapes. Notably, kinase inhibitors induced inhibitor- and patient-specific off-target effects and pathway crosstalk, highlighting the non-genetic heterogeneity of CRC PDOs. The authors suggested that Kinase signaling rewiring was only modestly affected by mutations, indicating that non-genetic mechanisms contribute significantly to therapy resistance. Moreover, upregulation of stemness and differentiation genes was observed upon kinase inhibitor treatment, providing insights into therapy-induced phenotypic changes in CRC. Imaging mass cytometry-based profiling of primary tumors further revealed spatial heterocellular crosstalk and tumor-immune interactions within the TME. These findings establish a framework for integrating tumor cell-intrinsic signaling with external TME cues to inform precision oncology and immunotherapy in CRC (Plattner et al., 2023). Another study identified SERPINC1 as a key gene associated with CRC liver metastasis using transcriptomic data and immunohistochemical analysis from CRC patient tissues (Le et al., 2024). High SERPINC1 expression was significantly linked to advanced TNM stage and poor 5-year survival in CRC patients. Functional assays, including colony formation, CCK-8, and transwell migration, demonstrated that SERPINC1 promotes malignant proliferation and metastasis of CRC cells through TGF-β1-mediated epithelial-mesenchymal transition (EMT) (Le et al., 2024). Furthermore, higher SERPINC1 expression was associated with reduced sensitivity to immune checkpoint therapy, suggesting that targeting SERPINC1 could provide a novel therapeutic strategy for patients with CRC liver metastases (Le et al., 2024).

Furthermore, PDO models have been used to uncover mechanisms driving tumor evolution and drug resistance in BRCA1-mutant ovarian cancer (Xie et al., 2024). Recent findings revealed that BRCA1 promotes ferroptosis by catalyzing K6-linked polyubiquitination and degradation of GPX4. Loss of BRCA1 increases GPX4 levels, leading to ferroptosis resistance. PDO-based studies demonstrated that combining PARP inhibitors (PARPi) with a GPX4 inhibitor yielded synergistic anti-tumor effects in BRCA1-deficient ovarian cancer PDOs, underscoring GPX4 as a promising therapeutic target for BRCA1-mutant cancers (Xie et al., 2024). Collectively, these findings underscore the utility of PDOs as robust preclinical models to elucidate tumor evolution, intratumoral heterogeneity, and mechanisms of therapy resistance. However, standardization of culture methods and long-term reproducibility remain important barriers to translating PDO-based heterogeneity studies into the clinic.

### 3.3 PDO-based immuno-oncology models

**FIGURE 3:**
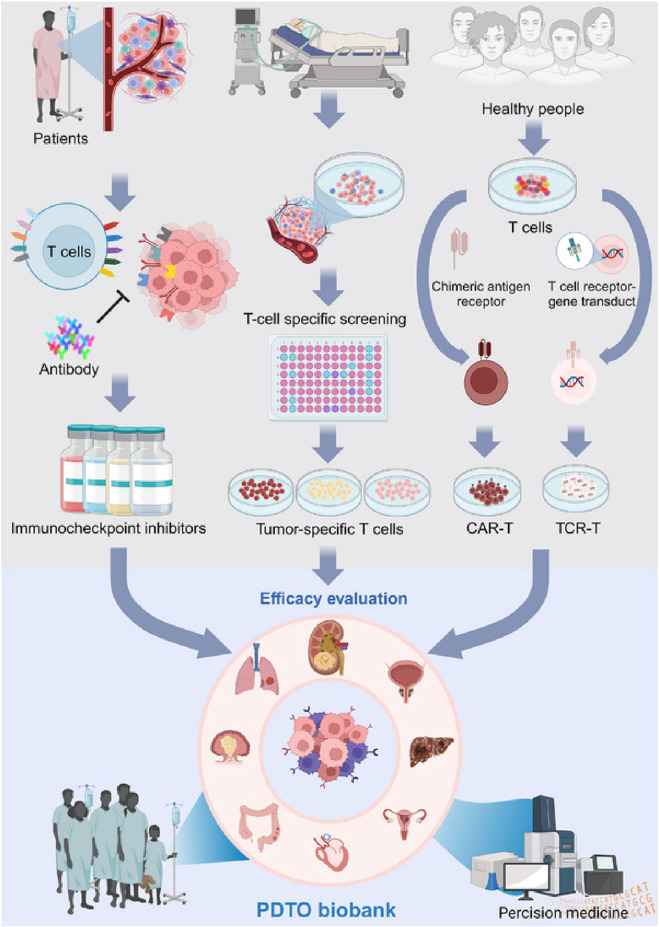
Patient-derived tumor organoids (PDTOs) act as models for forecasting immunotherapy outcomes, including ICI and immune cell therapies. Effective PDTOs should incorporate various cell types, particularly immune cells, and accurately reflect the TME to ensure reliable testing. Adapted with permission from Mei et al. (2024).

The human immune system employs multiple defense mechanisms, including humoral immunity (mediated by antibodies) and cell-mediated immunity (involving immune cells such as T cells), to eliminate tumor cells. However, these mechanisms are often suppressed within the TME, leading to immune escape and tumor progression. Several factors in the TME contribute to this immune suppression, including hypoxia, epigenetic modifications, and translational regulation. These modifications create an environment that allows cancer cells to evade immune detection and continue to grow and spread (Ringquist et al., 2021). The complex interplay between malignant cells and surrounding non-malignant components within the TME also influences key cancer-related processes such as tumor progression, metastasis, carcinogenesis, and drug resistance. Therefore, accurately modeling these interactions is crucial for enhancing the effectiveness of immunotherapies (Figure 3).

**FIGURE 4:**
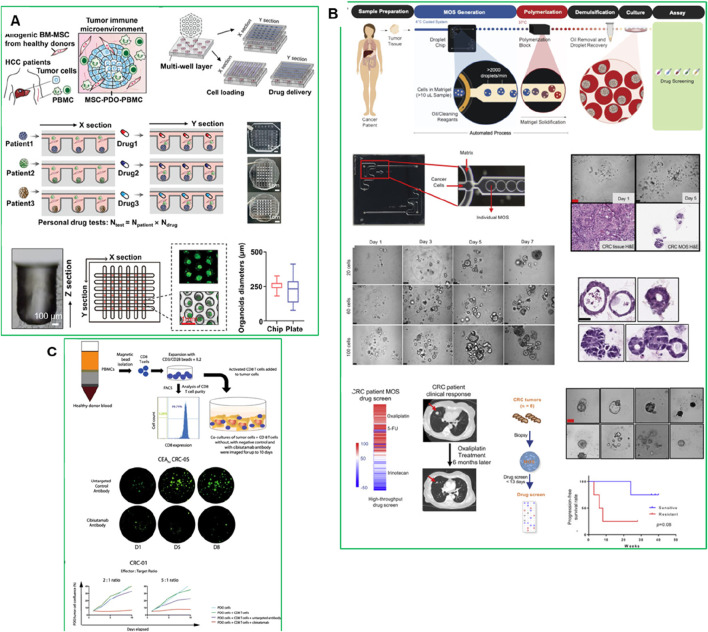
**(A)** Establishment of an HCC-TME using PDO co-cultured with MSC and PBMC on a high-throughput microfluidic chip (Zou et al., 2023). **(B)** Establishing CRC MOS for drug screening and clinical validation (Ding et al., 2022). **(C)** PDO and CD8^+^ T cell co-culture model with cibisatamab treatment for functional assessment. Adapted with permission.

PDOs are also playing an increasingly important role in the field of immunotherapy (Zhang et al., 2022). They have been used to model patient-specific responses to immune checkpoint inhibitors and CAR-T cell therapies, offering a platform to study the interaction between tumors and the immune system. PDO-based immuno-oncology models allow the co-culture of PDOs with autologous immune cells, including T cells, dendritic cells, and natural killer (NK) cells. This creates a more physiologically relevant environment, allowing researchers to better predict how a patient’s tumor will respond to immunotherapy. For instance, PDO models have been used in studying immunotherapy responses in hepatocellular carcinoma (HCC). A recent study developed an HCC organoid-on-a-chip platform by co-culturing HCC-PDOs with mesenchymal stromal cells (MSC), PBMC, and cancer-associated fibroblasts (CAFs) to mimic the TME (Figure 4A) (Zou et al., 2023). This model increased PDO success rates, accelerated growth, and enhanced immune cell survival and differentiation into tumor-associated macrophages. The microfluidic chip enabled high-throughput drug screening and accurately predicted patient responses to anti-PD-L1 drugs, offering a valuable platform for optimizing HCC immunotherapy (Zou et al., 2023). While PDO-immune co-culture platforms have shown promise, challenges remain regarding immune compatibility and long-term maintenance. Organoids transplanted into animal models often lack autologous immune context, leading to false-negative predictions for immunotherapies. Moreover, even *in vitro* co-cultures with PBMCs or TILs are typically limited to short-term assays due to immune cell exhaustion. Developing autologous or engineered immune-compatible platforms will be essential to improve predictive power in immuno-oncology research.

Recently, micro-organospheres (MOSs) have also emerged as a more rapid and clinically adaptable platform for immunotherapy testing. The integration of genomic profiling and biomarker analysis with PDO and MOS models could further enhance the predictive accuracy and clinical relevance of immuno-oncology treatments (Jain, 2021). For instance, MOSs were generated using droplet emulsion microfluidics with temperature control and dead-volume minimization, allowing for the creation of thousands of MOSs from small biopsy samples within 14 days-a timeline suitable for guiding clinical decisions (Figure 4A). Unlike traditional PDOs, MOSs maintain the original TME, including stromal and immune components, and allow T cell infiltration, making them highly suitable for testing immuno-oncology therapies. A clinical study in metastatic colorectal cancer (CRC) demonstrated that MOS-based drug screening accurately predicted patient tumor responses to immunotherapies such as PD-1 blockade, bispecific antibodies, and T cell therapies (Figure 4B) (Ding et al., 2022). MOSs showed high predictive accuracy for drug response in metastatic CRC patients, correlating with clinical outcomes. The platform also enabled testing of immune-oncology therapies such as PD-1 blockade, T cell therapies, and bispecific antibodies (Ding et al., 2022).

PDOs have also been used to study resistance to bispecific antibodies. Gonzalez-Exposito et al. (2019) demonstrated that PDOs from multidrug-resistant metastatic CRC could predict sensitivity to the bispecific antibody cibisatamab, which targets carcinoembryonic antigen (CEA) and CD3 on T cells (Figure 4C) (Gonzalez-Exposito et al., 2019). PDOs were classified into CEA_hi_ (n = 3), CEA_lo_ (n = 1) and CEA_mixed_ PDOs (n = 4), that stably maintained populations of CEA_hi_ and CEA_lo_ cells, and mixed CEA expression groups. CEA_hi_ PDOs were sensitive to cibisatamab, whereas CEAlo PDOs were resistant due to activation of the WNT/β-catenin pathway. Inhibition of this pathway restored CEA expression and increased sensitivity to cibisatamab, highlighting the potential for combining WNT/β-catenin inhibitors with bispecific immunotherapies (Gonzalez-Exposito et al., 2019). This study demonstrates the value of PDO-based T cell co-culture models for identifying resistance mechanisms and optimizing combination therapies (Gonzalez-Exposito et al., 2019). Thus the PDO-based models have demonstrated the ability to replicate the immune landscape of individual tumors, providing an accurate platform for testing these therapies.

## 4 PDO-based microfluidic and biomimetic platforms

Embedding PDOs into microfluidic chips, have further enhanced cancer research by enabling real-time studies of TME dynamics. These “organ-on-chip” (OOC), multi-organ-on-a-chip (MoC), “patient-on-chip” (POC) systems allow for the investigation of complex biological processes, including immune cell infiltration and stromal remodeling (Sood et al., 2023). These models employing PDOs have emerged as a promising alternative technology for testing and developing TCTs (Palaniyandi et al., 2024). However, compared to conventional PDO cultures, which provide scalability and high-throughput screening capacity, microfluidic and biomimetic systems offer superior physiological relevance, vascular integration, and dynamic cell–cell interactions but are limited by lower throughput, higher technical demands, and cost. Thus, these platforms are complementary rather than fully substitutive. Recently, a lot of studies showed promising results using these models, some of them we discussed in following sections.

### 4.1 Organ-on-chip

These microfluidic devices are designed to mimic human tissues and organs on a smaller scale, replicating key dynamic processes that occur *in vivo*. By incorporating human cancer cells, specifically PDOs, within the chip’s microchannels and introducing dynamic flow conditions, researchers can create biomimetic cancer-on-a-chip (CoC) models (Cavero et al., 2019). These models closely resemble real TME. Furthermore, integrating patient-derived cells into these systems enables the development of more personalized and precise therapeutic strategies. Recently, Sun et al. (2025) demonstrated the potential of using microfluidic OOC systems combined with PDOs for the development and evaluation of novel OVs. They developed a recombinant oncolytic adenovirus, AD4-GHPE, and evaluated its efficacy in hypopharyngeal and breast cancer organoids using OOC systems. AD4-GHPE showed three distinct antitumor mechanisms: tumor-specific cytotoxicity, reduced programmed death ligand 1 (PD-L1) expression to increase CD8^+^ T-cell activity, and granulocyte–macrophage colony-stimulating factor (GM-CSF) secretion (Sun et al., 2025).

**FIGURE 5:**
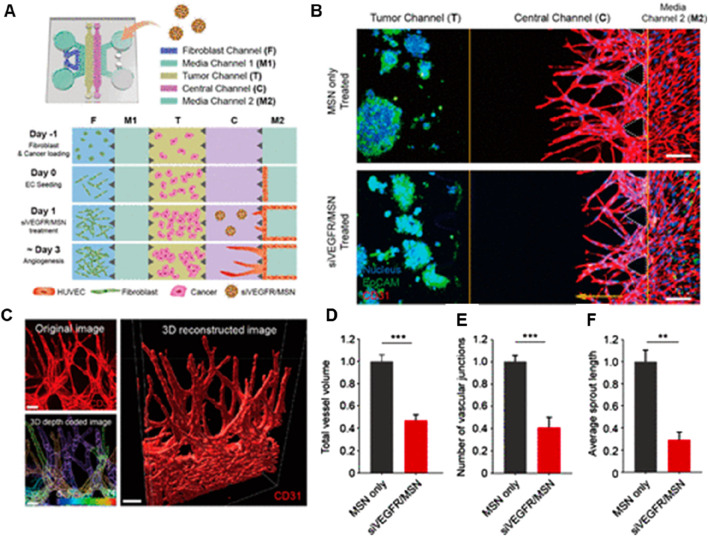
3D microfluidic platform for *in vitro* cancer angiogenesis regulation using siVEGFR/MSN treatment. **(A)** Schematic of chip design and cell loading sequence. **(B)** Representative confocal 3D images showing HepG2 angiogenesis with or without siVEGFR/MSN treatment. **(C)** 3D reconstructed image of sprouting; depth-coded showing sprouts at different depths. **(D–F)** Quantitative analysis of vessel volume, sprout length, and vascular junctions (Lee et al., 2021). Adapted with permission.

OOC models have also been explored for targeting angiogenesis, which refers to the formation of new blood vessels that supply nutrients and oxygen to tumors, enabling them to grow and spread. Targeting these angiogenic pathways is a common strategy in cancer therapy to restrict tumor blood supply and inhibit growth. Lee et al., 2020 showed the potential of RNAi-based nanomedicine targeting angiogenic pathways using OOC models (Figure 5) (Lee et al., 2021). However, previous *in vitro* and *in vivo* models were limited in evaluating complex 3D angiogenic morphology. To address this, authors have developed a 3D microfluidic cancer angiogenesis model, which enables precise visualization of directional 3D angiogenic sprouting toward cancer cells. The integration of 3D imaging and tissue clearing technology further enhances the evaluation of tumor vessel normalization and anti-angiogenic effects, offering a more accurate and biomimetic platform for testing therapeutics (Lee et al., 2021).

### 4.2 Multiorgan-on-a-chip (MoC)

**FIGURE 6:**
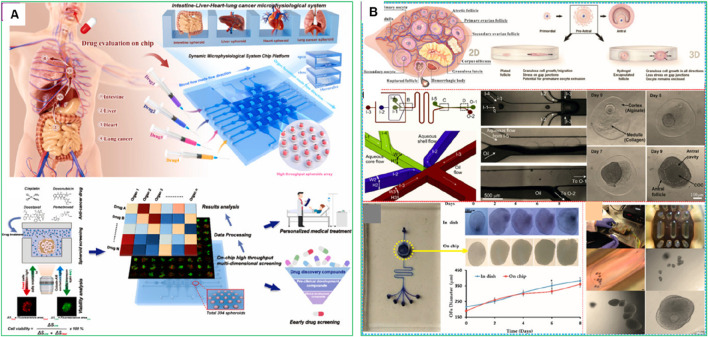
Microphysiological System Chip Platform (MSCP) for high-throughput drug screening and microphysiological system construction. **(A)** Schematic of drug absorption from the intestine to vital organs (liver, heart, lung, intestine), mimicked by the MSCP to evaluate multiple drugs simultaneously (Zhu et al., 2024). **(B)** Overview of the microfluidic chips that have been used to model fallopian tubes and the uterus (Yan et al., 2023). Adapted with permission.

While single-OOC models have proven effective in replicating *in vivo* conditions, they fall short in capturing organ-to-organ interactions, which are crucial for studying cancer metastasis and systemic drug toxicity. To address this limitation, multi-organ-on-a-chip (MoC) systems have been developed, providing a more comprehensive platform for mimicking complex physiological environments. These MoC models have been particularly valuable in investigating cancer metastasis, cell migration, and invasion into secondary organs, often referred to as metastasis-on-a-chip platforms (Del Piccolo et al., 2021). For instance, metastatic cells undergo complex signaling events as they detach from the primary tumor, travel through the bloodstream or lymphatic system, and colonize secondary organs. MoC platforms enable the real-time tracking of these processes, providing valuable insights into how cancer cells adapt to various microenvironments and evade immune surveillance. Notably, MoC systems have successfully integrated up to fifteen interconnected organs, offering an advanced approach to evaluating the targeting efficiency and potential off-target effects of anticancer therapies (Zhu et al., 2024). Moreover, MoC systems enable the assessment of systemic toxicity and drug resistance by simulating the interactions between the liver, kidney, and other metabolically active tissues involved in drug metabolism and clearance (Ozer et al., 2023). For instance, Zhu et al. recently introduced a Microphysiological System Chip Platform (MSCP) designed for high-throughput, parallel drug testing using a lung cancer spheroid model and a multi-organ (intestine-liver-heart-lung) system (Figure 6A). The MSCP allowed for real-time assessment of drug efficacy and side effects through fluid-based physiological communication, simulating drug absorption and distribution across organs. This platform represents a significant step toward more precise disease modeling and personalized drug development (Zhu et al., 2024). In another study, Dornhof et al. (2022) also developed a microfluidic organ-on-chip platform with integrated sensors to monitor oxygen, lactate, and glucose in real-time. It supported the growth of patient-derived triple-negative breast cancer organoids under control conditions, enabling continuous, quantitative monitoring of drug responses and metabolic changes (Dornhof et al., 2022).

Advanced MoC models also facilitate the testing of combination therapies and immune-based treatments in a physiologically relevant context. By incorporating immune cells, endothelial cells, and stromal components into the platform, researchers can explore how the immune system responds to targeted therapies and immunotherapies. For instance, Yan et al. (2023) highlighted that microfluidic chips have transformed the understanding and management of female reproductive health by simulating complex physiological and pathological conditions (Figure 6B) (Yan et al., 2023). These platforms have been used to model the ovary, fallopian tube, uterus, placenta, and cervix, enabling studies on follicle and oocyte culture, gamete manipulation, cryopreservation, and drug screening. MoC systems have also been applied to study endometriosis, ovarian, endometrial, and cervical cancers, providing valuable insights for improving therapies and diagnostic approaches. However, these microfluidic platforms lacked the ability to replicate the complex vascular network and dynamic blood flow present *in vivo*. This limited nutrient and oxygen delivery, waste removal, and immune cell interaction, reducing the physiological relevance of the models. Vessel-on-a-chip technology addresses these gaps by integrating vascular structures, improving tissue viability, and enabling more accurate drug testing and disease modeling.

### 4.3 Vessel-on-a-chip

**FIGURE 7:**
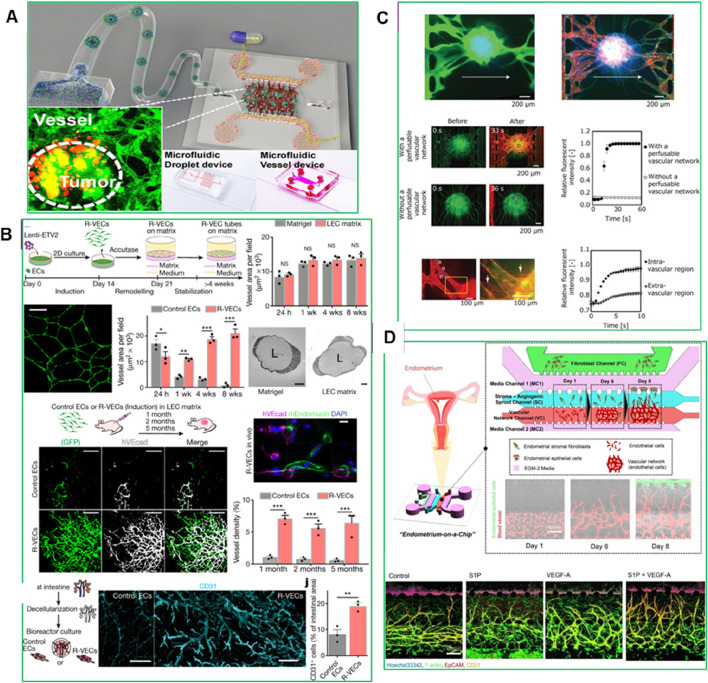
**(A)** Experimental set-up for vessel formation (Wu et al., 2023), **(B)** The ‘reset’ vascular endothelial cells (R-VECs) self-assemble into 3D durable vessels *in vitro* and *in vivo* (Palikuqi et al., 2020), **(C)** Perfusion of the interior of the spheroid using the constructed vascular network (Nashimoto et al., 2017), **(D)** Microfluidic Device for 3D Vascularized Endometrial Model: Schematic representation and confocal images illustrating the reconstitution of a natural endometrial microenvironment using a microfluidic 3D tri-culture system. The model incorporates endometrial stromal fibroblasts, epithelial cells, and endothelial cells, effectively replicating the structural and functional complexity of the native endometrium (Ahn et al., 2021). Adapted with permission.

Vessel-on-a-chip models have been employed to investigate the behavior of drugs within the tumor microcirculation system. By replicating the structural and molecular characteristics of tumor-associated blood vessels, these models enable the assessment of drugs transport, adhesion, and penetration under physiological flow conditions (Caballero et al., 2017). For instance, Wu et al. (2023) demonstrated the use of gelatin-methacryloyl (GelMA) hydrogel to mimic the ECM, creating a more physiologically relevant TME. In their study, HCT-116 tumor cells were encapsulated into micro-GelMA beads using a microfluidic droplet technique, which allowed for the recreation of tumor–stromal interactions by incorporating human lung fibroblasts. This resulted in the formation of a core–shell heterotypic tumor structure that closely mimicked the native TME (Wu et al., 2023). The cell-laden beads were then integrated into a functional on-chip vessel network platform, restoring key tumor–tumor and vascular interactions. When paclitaxel was tested on this vessel-supported model, the researchers observed increased drug resistance due to the vascularized TME, highlighting the model’s potential to improve the predictive accuracy of preclinical drug discovery (Figure 7A) (Wu et al., 2023).

A recent advancement in vessel-on-a-chip models is the Organ-On-VascularNet platform, where endothelial cells are reset to adaptable, vasculogenic cells through transient reactivation of embryonic-restricted ETS variant transcription factor 2 (ETV2) (Palikuqi et al., 2020). These cells self-organize into durable, branching vascular networks capable of transporting human blood and directly interacting with co-cultured organoids and tumoroids without the need for synthetic membranes. This adaptive vascular niche conforms to the specific characteristics of different tissues, enhancing the physiological relevance of PDO-based models for drug testing and TME studies (Figure 7B) (Palikuqi et al., 2020). Nashimoto et al. (2017) introduced a method to create a three-dimensional cellular spheroid with a perfusable vascular network in a microfluidic device. By defining cellular interactions between human lung fibroblasts (hLFs) in a spheroid and human umbilical vein endothelial cells (HUVECs) in microchannels, angiogenic sprouts were induced to form from the microchannels toward the spheroid, resulting in a continuous lumen (Figure 7C) (Nashimoto et al., 2017). This perfusable network allowed direct delivery of nutrients and biological substances to the spheroid’s interior, improving cell viability and mimicking the density and function of native tissue. This advancement enhances the potential for long-term tissue culture and drug screening, making vessel-on-a-chip systems more reliable for replicating *in vivo*-like drug responses.

Another example is developed by Ahn et al. (2021), a microengineered vascularized endometrium-on-a-chip model that accurately replicates the human endometrial microenvironment, consisting of three distinct layers: epithelium, stroma, and blood vessels, embedded within a 3D ECM in a spatiotemporal manner. This model successfully mimics key features of *in vivo* endometrial vasculo-angiogenesis and hormonal responses, displaying characteristics of both the proliferative and secretory phases of the menstrual cycle. Ahn et al. demonstrated the model’s utility in drug testing by evaluating the effects of the emergency contraception drug levonorgestrel, which induced increased endometrial permeability and blood vessel regression in a dose-dependent manner (Figure 7D) (Ahn et al., 2021). Furthermore, they provided a proof of concept for using the model to study embryo implantation, *in vitro* drug screening and discovery, offering a personalized platform for studying female reproductive health issues, including endometriosis, uterine cancer, and infertility (Ahn et al., 2021).

## 5 Engineering functional biomaterials to advance PDO systems

A central bottleneck in PDO technology is the inability to fully recapitulate the complex TME with high reproducibility and translational relevance. Functional biomaterials spanning natural ECM substitutes, synthetic hydrogels, nanoclays, and bioactive composites are now being engineered not only to provide structural and biochemical support but also to directly address these shortcomings. By enabling precise control over stiffness, degradability, ligand presentation, and bioactivity, these next-generation materials improve reproducibility, mechanical stability, and the fidelity of tumor–stroma–immune interactions within PDOs (Yi et al., 2021). Matrigel® is one such example, applied widely in PDO advancement. Two Matrigel is a widely used mouse-derived basement membrane extract that provides a supportive ECM-like environment for organoid growth and differentiation. A recent study used Matrigel to develop a human fallopian tube (HFT) organoid model from stem cells isolated from the isthmus and ampulla regions (Gatimel et al., 2025). The apical compartment of the HFT organoid supported significantly higher sperm motility compared to commercial fertilization media. After 48 h, progressive sperm motility in the HFT organoid was 31% ± 17 in the ampulla and 29% ± 15 in the isthmus, compared to 15% ± 15 in commercial media (P < 0.05). Even after 96 h, motility remained at ∼12%–13% in the HFT organoid while it was nearly undetectable in other conditions. This highlights the ability of Matrigel-based models to replicate functional reproductive environments (Gatimel et al., 2025). Additionally, Matrigel and other natural ECM substitutes fail to replicate the biomechanical and biochemical complexity of the human TME. Tumors *in vivo* experience complex mechanical forces, biochemical signaling, and dynamic cell-cell and cell-matrix interactions that are difficult to reproduce using traditional ECM substitutes. As a result, PDO models cultured in Matrigel often fail to capture the full complexity of tumor progression, metastasis, and therapy resistance. However, composite PEG-Matrigel™ hydrogels have also emerged as promising alternatives for studying tumor cell dissemination. Beck et al. designed a PEG-Matrigel™ composite hydrogel with tunable mechanical properties and adhesive peptide density, incorporating RGD motifs to enhance cell adhesion and signaling. Interestingly, their study revealed that mammary tumor organoids preferentially disseminated into Matrigel™, even in the absence of collagen I, suggesting that tumor progression and metastasis could be driven by physicochemical matrix properties rather than collagen-specific signaling. This challenges the traditional understanding of the role of collagen I in tumor progression and provides insight into new therapeutic strategies targeting matrix properties rather than specific signaling pathways (Cruz-Acuna and Garcia, 2019). While Matrigel® has traditionally served this purpose of better cell-matrix interactions, support tissue-specific signaling, and enhance the mechanical stiffness, its animal origin, batch variability, and undefined composition have prompted the development of alternative synthetic and bioengineered matrices.

### 5.1 ECM substitutes and functional hydrogel scaffolds

**FIGURE 8:**
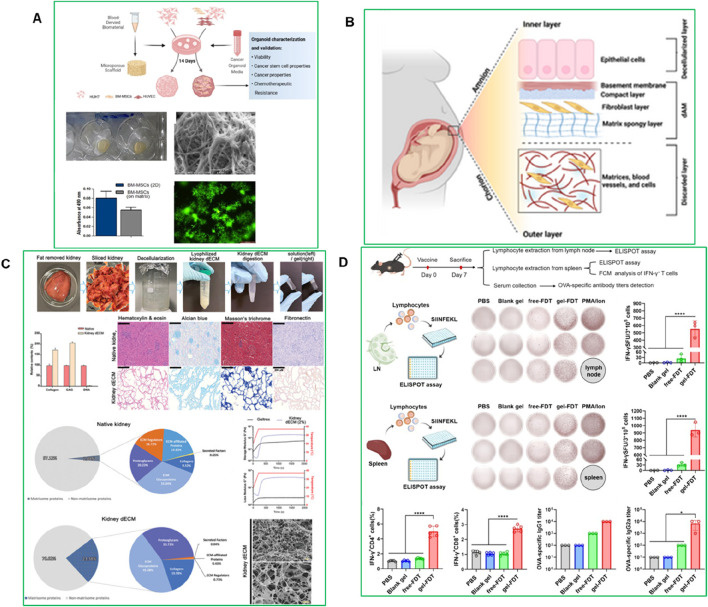
**(A)** Plasma-derived ECM characterization (El-Derby et al., 2024) **(B)** Structure of the human amniotic membrane (AM) used for developing HCC organoid models. The AM consists of an inner layer with epithelial cells, a basement membrane, and a fibroblast layer, followed by a matrix spongy layer (Atta et al., 2025). **(C)** Decellularization and characterization of porcine kidney tissue (Kim et al., 2022). **(D)** The gel-FDT induced a stronger antigen-specific immune response (Zhang et al., 2024).

The ECM provides not only structural support but also crucial biochemical and biomechanical cues essential for organoid development, differentiation, and function. To replicate these complex *in vivo* environments, diverse natural and synthetic hydrogels have been engineered as ECM substitutes in PDO systems. These scaffolds are being increasingly refined to enhance tunability, mechanical integrity, and biological relevance. A recent study developed a plasma-rich platelet ECM-based system for culturing HCC organoids, providing a more physiologically relevant and cost-effective platform for liver cancer modeling (El-Derby et al., 2024). Organoids were generated from HUH-7 hepatoma cells cultured alone (homogeneous) or with mesenchymal stromal cells and endothelial cells (heterogeneous). The heterogeneous organoids exhibited enhanced invasion potential, cancer stem cell populations, and late-stage HCC genetic signatures compared to homogeneous models. This plasma ECM-based approach improves organoid viability, cancer properties, and chemoresistance, offering a scalable and standardized platform for HCC drug screening and pathogenesis studies (Figure 8A) (El-Derby et al., 2024).

Another study developed a hepatocellular carcinoma (HCC) organoid model using a decellularized human amniotic membrane (dAM) as a scaffold combined with Huh-7 cells, bone marrow mesenchymal stromal cells (BM-MSC), and human umbilical vein endothelial cell-conditioned medium (HUVEC-CM) (Figure 8B) (Atta et al., 2025). The organoid maintained structural integrity and viability for over 21 days, showing increased angiogenic activity through VEGF expression and a metabolic shift toward glycolysis, reduced oxidative phosphorylation, and altered urea cycle progression. This dAM-based model effectively replicates the HCC TME and metabolic landscape, providing a promising platform for studying tumor progression and testing targeted therapies (Atta et al., 2025). Hydroxypropyl cellulose-based hydrogels have shown promising results in generating human tumor organoids from hepatocellular carcinoma (HCC)-derived PDX lines. Similarly, Fong et al. developed an *in vitro* system using hydroxypropyl cellulose hydrogels conjugated with galactose ligands, which supported the viability, proliferation, and intra-tumoral heterogeneity of HCC-PDX organoids (Fong et al., 2018). These hydrogels offer *in vivo*-like mechanical stiffness, enable spheroid size control, and prevent inner core cell death caused by diffusion limitations issues often seen in conventional matrices like collagen I. Importantly, the hydrogel showed minimal drug absorption, making it suitable for high-throughput drug screening. The organoids generated using this platform exhibited a strong genomic and transcriptomic resemblance to their *in vivo* counterparts and retained intra-tumoral heterogeneity. Moreover, the organoids demonstrated sensitivity to drugs typically used for HCC patients, confirming the predictive value of this system for pre-clinical drug development. However, the physical constraint exerted by the non-degradable hydrogel crosslinks may have limited the proliferation of organoids, highlighting the need to incorporate matrix metalloproteinase (MMP)-sensitive sites to allow for matrix remodeling and better mimic *in vivo* tumor growth.

In parallel, several next-generation biomaterials are emerging to overcome the limitations of traditional ECM substitutes. For instance, Norbornene-functionalized hyaluronic acid (NorHA) hydrogels offer tunable stiffness and degradability, making them ideal for studying immune cell infiltration and tumor–immune interactions (Cruz-Acuna et al., 2023). Silk fibroin-based hydrogels provide excellent biocompatibility, mechanical strength, and sustained drug release capacity, supporting long-term organoid culture and regenerative modeling (Liu et al., 2022). Pluronic® F127, a thermo-responsive hydrogel, enables injectable organoid delivery and reversible encapsulation, ideal for transplantation and dynamic modeling (Chatterjee et al., 2019; Wang et al., 2024). Emerging conductive polymers such as PEDOT:PSS and polypyrrole are being integrated into PDO platforms for real-time monitoring of drug responses and cell signaling *via* electrical readouts (Song et al., 2024). Moreover, MXenes, a novel family of 2D nanomaterials, are gaining traction for their utility in photothermal therapy, biosensing, and drug delivery due to their high conductivity, biocompatibility, and tunable surface chemistry (Liu et al., 2018; Scheibe et al., 2019). Collectively, these advanced biomaterials are expanding the functional and translational capacity of organoid models, enabling more precise control of the TME and facilitating high-throughput, clinically relevant applications.

While ECM-based models replicate key structural and biochemical cues, synthetic biomaterials offer enhanced control over biomechanical properties, enabling greater precision in drug screening and tissue modeling. However, Matrigel® poses several translational challenges beyond batch variability. As a tumor-derived, animal-based matrix, it contains undefined components and residual growth factors, introducing biological noise and potential tumorigenic risks. Its lack of FDA approval and incompatibility with GMP processes limit its clinical translation. Emerging xeno-free alternatives such as VitroGel®, PEG hydrogels, and synthetic nanocellulose scaffolds (e.g., GrowDex®) offer reproducibility and tunability better suited for regulatory environments (Table 2).

**TABLE 2:** Summarizes the biomaterials discussed in this section, outlining their properties, advantages, and applications in PDO systems.

Biomaterial	Type	Origin	Used in	Key advantages	Limitations	Applications in PDOs
Matrigel®	Natural ECM hydrogel	Mouse sarcoma (BME)	Organoids, co-cultures	Biochemical richness, supports differentiation	Batch variability, undefined, animal-derived	Widely used for PDO growth and TME modeling
PEG	Synthetic polymer	Fully synthetic	Drug screening, stiffness modeling	Tunable stiffness, reproducible, inert	Requires functionalization for adhesion	Tumor stiffness mimicry, HTS
Laponite	Synthetic nanoclay	Synthetic	Drug delivery, TME mimicry	pH/temperature responsive, injectable	May require blending for structure	Controlled drug release, hypoxic TME simulation
NorHA	Synthetic hydrogel	Modified hyaluronic acid	Immune cell co-culture	Tunable degradation/stiffness, bioactive	Needs photo-initiation	T cell infiltration, immune–tumor interaction models
GelMA	Hybrid hydrogel	Gelatin-derived	3D bioprinting, vascular organoids	Biocompatible, photo-crosslinkable	UV exposure and gelation complexity	Tumor vascularization, regenerative models
VitroGel®	Synthetic hydrogel	Xeno-free, commercial	HTS, clinical testing	Ready-to-use, reproducible, clinical-grade	Cost, proprietary formulation	Screening, personalized medicine
GrowDex®	Natural hydrogel	Nanocellulose (plant)	Biobanking, personalized models	Animal-free, injectable, ethical	Lacks complex ECM cues	Organoid preservation, ethical models
Collagen I/IV	Natural ECM proteins	Human/animal-derived	Co-culture, invasion models	Physiological adhesion, immune relevance	Not highly tunable mechanically	Stromal co-cultures, angiogenesis studies
PuraMatrix™	Self-assembling peptide hydrogel	Synthetic	Neural/cancer organoids	Animal-free, defined, supports 3D growth	Expensive, soft gel	Neural organoids, cancer PDO architecture
Pluronic® F127	Thermo-responsive hydrogel	Synthetic	*In vivo* delivery, dynamic modeling	Injectable, reversible gelation	Poor bioactivity without additives	Organoid transplantation, dynamic systems
Silk fibroin	Natural protein hydrogel	Silkworm-derived	Long-term culture, drug release	Biocompatible, slow degradation	More complex to fabricate	Sustained drug delivery, structural PDO support
MXenes	2D nanomaterials	Synthetic	Biosensing, photothermal therapy	Conductive, high surface area, biofunctionalizable	Needs composite formulation	Real-time monitoring, photothermal PDO models
PEDOT:PSS/ Polypyrrole	Conductive polymer	Synthetic	Biosensing, electro-responsive platforms	Conductivity, real-time readouts	Non-biodegradable, fabrication complexity	Monitoring cell response, HTS-integrated PDOs

### 5.2 Synthetic biomaterials: PEG, laponite, and alginate systems

Synthetic biomaterials, such as Laponite, polyethylene glycol (PEG), and alginate derivatives, offer precise control over matrix composition, stiffness, porosity, and biochemical cues. These materials can be engineered to mimic the biomechanical properties of the native tumor environment, allowing for better replication of the physical forces and signaling gradients present in human tumors. For example, PEG-based hydrogels can be tuned to match the stiffness of specific tumor tissues, ranging from soft tissues like breast and pancreatic cancer to stiffer solid tumors like bone metastases. This level of mechanical control improves the relevance of PDO-based drug screening, as studies have shown that matrix stiffness influences cancer cell proliferation, invasion, and therapy resistance. A recent study developed a bioink combining gelatin, alginate, and liver decellularized extracellular matrix (LdECM) for 3D bioprinting (You et al., 2024). The bioink enhanced bone mesenchymal stem cell (BMSC) proliferation and differentiation, and *in vivo* tests showed improved angiogenesis and bone regeneration in a rat model (You et al., 2024). Another study, demonstrated that culturing kidney organoids derived from human pluripotent stem cells (hPSCs) in a kidney decellularized extracellular matrix (dECM) hydrogel enhanced vascularization and glomerular development. Single-cell transcriptomics showed that vascularized kidney organoids exhibited more mature glomerular structures and greater similarity to human kidneys than those cultured without dECM. This approach also enabled modeling of Fabry nephropathy and improved vascular integrity after transplantation into mouse kidneys. This highlights the potential of dECM-based scaffolds to improve organoid complexity and functionality for disease modeling and regenerative medicine (Figure 8C) (Kim et al., 2022). Laponite-based biomaterials are also widely used, because they are pH- and temperature-responsive, enabling dynamic drug release under conditions that mimic the acidic and hypoxic TME. This allows for sustained drug exposure and improved prediction of therapeutic efficacy. A recent study introduced a Laponite-based gel-vaccine platform with self-adjuvanting properties, designed for sustained antigen delivery and immune cell recruitment (Figure 8D) (Zhang et al., 2024). This system enhanced both humoral and cellular immune responses and demonstrated significant therapeutic efficacy across multiple tumor models, including complete tumor eradication in a murine colorectal peritoneal metastasis model following a single dose (Zhang et al., 2024).

In addition to PEG and alginate-based systems, several other biomaterials have emerged as promising alternatives or complements to Matrigel®. Gelatin methacrylate (GelMA) is a photo-crosslinkable, tunable hydrogel widely used in 3D bioprinting and vascularized tumor models due to its excellent biocompatibility and structural stability (Zhou et al., 2023b; Xiao et al., 2019). VitroGel®, a xeno-free, ready-to-use hydrogel, offers reproducible performance, tunable stiffness, and compatibility with HTS, making it suitable for clinical and pharmaceutical applications (Zhang et al., 2025; Borges et al., 2023). GrowDex®, a plant-based nanocellulose hydrogel, is fully animal-free and has gained popularity for its ease of use and ethical advantages in personalized medicine and biobanking (Walz et al., 2023). Fibrin gels, derived from fibrinogen, are natural and biodegradable, commonly used for modeling tumor angiogenesis and co-culturing with endothelial or immune cells. Tumor-derived decellularized ECM (dECM) hydrogels provide cancer-type-specific biochemical and mechanical cues and have been used to better replicate the native TME in organoid cultures (Zhu et al., 2023). Lastly, an additional multifunctional biomaterial, BG-Mngel a manganese-doped bioactive glass hydrogel-has recently shown promise for melanoma therapy. Beyond structural support, it elicits potent anti-tumor immune responses *via* STING pathway activation, promotes angiogenesis, and facilitates wound regeneration post-surgery, especially when used in combination with immune checkpoint inhibitors like anti-PD-1 (Liu X. et al., 2024).

In addition to established systems, several advanced biomaterials have recently emerged with high relevance to PDO engineering. Zwitterionic hydrogels provide excellent anti-fouling and immune-evasive properties, making them ideal for co-culture and transplant models (Wang et al., 2025). Self-healing hydrogels, based on dynamic covalent or host–guest interactions, offer mechanical resilience and long-term stability (Pishavar et al., 2021). Micropatterned PEG-based hydrogels, often fabricated *via* 3D bioprinting or photolithography, allow spatial control of organoid architecture and mimic tissue zonation. Organ-specific decellularized ECMs (e.g., brain, pancreas, lung) enhance organoid fidelity by preserving tissue-specific cues (Tran et al., 2022). Additionally, DNA-based hydrogels and aptamer-functionalized matrices enable precise growth factor presentation and real-time biosensing (Wu et al., 2025). Together, these materials support complex, dynamic PDO environments for precision oncology and high-throughput functional screening. This type of immunomodulatory and regenerative biomaterial underscores the potential of next-generation hydrogels in not only supporting PDOs but also modeling tumor–immune dynamics and healing processes within a single platform (Liu W. S. et al., 2024).

Beyond enhancing structural fidelity and controlled drug delivery, functional biomaterials have also laid the groundwork for developing advanced co-culture platforms that better emulate the cellular heterogeneity of the TME. While tuning material properties are critical for replicating biomechanical and biochemical cues, integrating stromal and immune components within these engineered matrices is essential for capturing the dynamic, functional interactions that drive tumor progression. An example of such an immunomodulatory biomaterial is the ROD peptide hydrogel, comprising RADA16-I peptide, lysed OK-432, and doxorubicin, developed for treating residual hepatocellular carcinoma after incomplete radiofrequency ablation. This hydrogel exhibited a controlled drug release profile and robustly activated the STING pathway, promoting dendritic cell maturation and enhancing CD4^+^/CD8^+^ T cell infiltration while suppressing regulatory T cells (Cao et al., 2023). The study demonstrated long-term tumor suppression and immune memory formation, emphasizing the promise of peptide-based hydrogels in bridging immunotherapy and biomaterial-assisted cancer treatment. Additionally, it is crucial to comprehend how these biomaterials facilitate co-culture systems that replicate tumor–stroma–immune interactions.

### 5.3 Co-culture with stromal and immune cells

**FIGURE 9:**
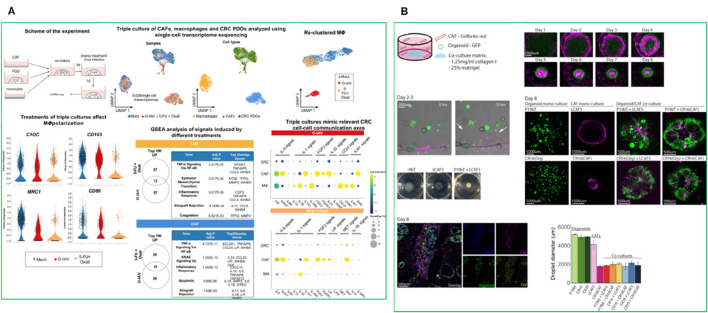
**(A)** Single-cell transcriptomics of the triple co-cultures revealed distinct myeloid cell states and gene expression signatures shaped by chemotherapy or oncolytic influenza A virus (O-IAV) treatment (Kabiljo et al., 2024). **(B)** Spontaneous reorganization of cancer cells and CAFs into macroscopic mini-tumors (Strating et al., 2023).

Functional biomaterials also enable the incorporation of bioactive ligands that facilitate cell-matrix interactions and tissue remodeling (Goodarzi and Rao, 2024). CAFs are key components of the TME that play a crucial role in tumor progression and therapy resistance. CAFs secrete ECM components, promote epithelial-to-mesenchymal transition (EMT), and create an immunosuppressive environment by releasing factors such as TGFβ1 and VEGFA (Sun et al., 2019). Incorporating CAFs into PDO models provides a more physiologically relevant platform for studying tumor-stroma interactions, immune evasion, and drug resistance. Recently, Kabiljo et al., 2024, showed the use of patient-derived colorectal cancer (CRC) organoids in triple co-culture systems that include CAFs and monocytes to model tumor-associated macrophages (TAMs) (Figure 9A) (Kabiljo et al., 2024). These systems successfully mimic TAM-like phenotypes and allow evaluation of treatment-induced immune modulation. Notably, chemotherapy induced pro-inflammatory macrophage polarization and enhanced phagocytic activity, especially in the presence of CAFs, offering a powerful *ex vivo* model for precision oncology applications (Kabiljo et al., 2024). Another study by Strating et al. (2023), in which the authors developed a co-culture system combining colon cancer PDOs with immortalized CAFs derived from liver metastases (Figure 9B). CAFs produced collagen IV, which supported glandular tumor formation and structural integrity, mirroring human cancer histology. Single-cell RNA sequencing revealed that CAFs induced EMT and promoted glycolysis, ECM remodeling, and hypoxia-related gene expression. This created an immunosuppressive environment with elevated levels of TGFβ1, VEGFA, and lactate, which inhibited T cell proliferation. This model closely resembles the mesenchymal-like CMS4 subtype of colon cancer and provides a valuable platform for studying tumor-stroma interactions and testing immunotherapies (Strating et al., 2023).

Hydrogel-based co-culture models have further expanded the physiological relevance of PDO platforms (Ng et al., 2019). For instance, Luo et al. developed a CRC-PDO co-culture model using a hyaluronan-gelatin hydrogel matrix, which better mimics the native TME (Luo et al., 2021). This model enabled the successful co-culture of CRC PDOs with patient-derived CAFs, which are known to contribute to tumor progression and drug resistance. The hydrogels maintained key molecular characteristics of the original patient tumors while sustaining both CRC PDO and CAF viability even without growth factor supplementation. Interestingly, the presence of CAFs restored distinct biological pathways that were absent in PDO culture alone, and thus proved effective for evaluating standard-of-care drugs (Luo et al., 2021). In a related strategy, a biohybrid 3D hydrogel system composed of matrix metalloproteinase-degradable PEG–heparin was functionalized with tumor-relevant ECM-derived peptides, including RGD, GFOGER (from collagen I), and IKVAV (from laminin-111), to investigate how biochemical cues from the TME affect cancer behavior (Taubenberger et al., 2016). This platform enabled controlled 3D co-culture of breast (MCF-7) and prostate (PC-3, LNCaP) cancer cells with endothelial and stromal cells. Notably, while less aggressive lines (MCF-7 and LNCaP) showed minimal changes, the invasive PC-3 cells displayed significantly enhanced invasiveness and endothelial infiltration when cultured in GFOGER- and IKVAV-modified hydrogels. These matrices induced a more malignant phenotype, simulating early events in cancer progression. This work highlights how defined, peptide-functionalized hydrogels can mechanistically dissect tumor–ECM–stroma interactions and support the development of more physiologically relevant cancer models (Taubenberger et al., 2016). Biomaterials functionalized with integrin-binding motifs, such as arginyl-glycyl-aspartic acid (RGD) peptides, enhance cell adhesion, migration, and proliferation within PDOs (Wijnakker et al., 2025). The ability to tune the biochemical signaling within the matrix creates a more physiologically accurate microenvironment. Furthermore, biosensor-integrated biomaterials enable real-time monitoring of PDO behavior and drug response (Liu H. et al., 2024). Conductive hydrogels allow for the measurement of electrical activity from cancer cells, providing insights into cell signaling dynamics and drug-induced cytotoxicity (Pan et al., 2021). Optical and fluorescent biomaterials, conjugated with quantum dots or fluorescent markers, enable real-time tracking of metabolic activity, apoptosis, and drug uptake within PDOs. Biomaterials embedded with pH-sensitive and oxygen-sensitive dyes offer additional insights into the metabolic state of the TME, facilitating the development of personalized therapeutic strategies (Kefayat et al., 2022). However, reproducibility and scalability remain major challenges for clinical translation. Patient-derived CAFs and immune cells introduce donor-specific variability, while complex hydrogel formulations hinder large-scale standardization. Overcoming these barriers will require xeno-free, modular biomaterials and automated technologies (e.g., 3D bioprinting, microfluidics) to reliably scale PDO co-cultures for drug screening and precision oncology. Altogether, incorporating co-culture systems into PDO models is crucial for accurately mimicking the TME, enabling more predictive studies of drug responses, immune interactions, and tumor progression in a patient-specific context.

## 6 PDOs in clinical translation

The refinement of PDO platforms through advanced biomaterials and co-culture strategies has accelerated their integration into clinical research. To ensure the reliability of organoid-based screening, quality control (QC) standards have been introduced in major biobanks. These include matching organoid and tumor mutational profiles (e.g., ≥90% SNV concordance), transcriptomic similarity *via* RNA-seq clustering, morphology scoring *via* histopathology, and pharmacologic response correlation. Adopting standardized QC protocols is essential for clinical implementation and reproducibility. Since 2020, there has been a significant rise in clinical trials investigating the use of PDOs for drug screening, personalized medicine, and disease modeling across various cancer types (Table 3). This surge in PDOs-based clinical trials reflects a growing recognition of their potential in overcoming inter- and intra-tumoral heterogeneity, improving treatment efficacy, and minimizing adverse effects. The first recorded clinical trial during this period, NCT04219137 (MOCHA), was initiated in January 2020 to investigate the molecular characteristics of gastroesophageal adenocarcinoma using organoid models. This marked the beginning of an era where organoids were increasingly employed in clinical oncology research. Shortly after, trials such as NCT04279509 (SCORE) were launched in February 2020 to test chemotherapy selection using high-throughput drug screening in PDOs for refractory solid tumors, including head and neck squamous cell carcinoma, colorectal cancer, breast cancer, and epithelial ovarian cancer. Similarly, NCT04371198 (May 2020) focused on establishing rectal cancer organoids to evaluate their role in disease modeling. Throughout 2020, several other trials expanded the scope of organoid applications. NCT04478877 (July 2020) aimed to establish and characterize meningioma PDOs through sequencing. In contrast, NCT04555473 (TAILOR), initiated in September 2020, combined sequencing and drug testing to evaluate longitudinal tumor progression in epithelial ovarian cancer. Trials such as NCT04611035 (Q-GAIN) and NCT04655573 investigated the predictive value of PDO-based drug screening in gastrointestinal and advanced breast cancers, respectively.

**TABLE 3:** Summary of clinical trials using PDOs across cancer types, highlighting key cancer types, trial numbers, drugs tested, predictive accuracy, and clinical relevance of PDOs-based screening.

Cancer type	Clinical trials (NCT IDs)	Drug tested	PDOs prediction accuracy	Clinical outcome
Colorectal Cancer	NCT04279509, NCT05304741, NCT05384184, NCT05640433	FOLFOX, irinotecan, cetuximab	High concordance with therapy response (>80%)	Improved prediction of resistance and therapy adaptation
Pancreatic Cancer	NCT05196334, NCT05351983	Gemcitabine + nab-paclitaxel, oxaliplatin	Validated predictive accuracy in matched patient cohorts	Early intervention guided by PDOs improved therapy alignment
Breast Cancer	NCT04655573, NCT05183425, NCT05007379, NCT06102824	Trastuzumab, pertuzumab, carboplatin	Strong correlation in HER2+ and triple-negative subtypes	Enhanced clinical matching in neoadjuvant trials
Lung Cancer	NCT05669586	Osimertinib, EGFR/ALK inhibitors	Effective at modeling resistance mutations (e.g., T790M)	Supported treatment modification strategies
Ovarian Cancer	NCT04279509, NCT04555473, NCT04768270, NCT05175326, NCT06085404	PARP inhibitors, taxane-platinum combinations	Moderate concordance; variable by BRCA status	Improved progression-free intervals in BRCA-mutated PDOs
Glioblastoma	NCT04865315, NCT04868396	Temozolomide, CAR-T	Low to moderate correlation	Promising platform; clinical translation ongoing
Gastric Cancer	NCT05203549	5-FU, platinum, PD-1 inhibitors	High correlation in early-stage PDO screens	Ongoing trials show potential for stratified therapy
Neuroendocrine Tumor	NCT04555473	Everolimus, somatostatin analogs	Limited validation; small sample sizes	Potential tool for individualized dosing regimens
Liver Cancer (HCC)	NCT05913141	Sorafenib, PD-1/PD-L1 inhibitors	Preliminary organoid-drug matching promising	Under clinical validation for use in second-line therapies
Biliary Tract/Cholangiocarcinoma	NCT05634694	FGFR inhibitors, immunotherapies	Under investigation	PDOs enable subtype-specific drug testing
Renal Cancer (VHL-related)	NCT06195150	Belzutifan, VEGFR inhibitors	Not yet validated	Exploratory use of PDOs in hereditary tumors
Glioblastoma/High-grade Astrocytoma	NCT04865315, NCT04868396	Temozolomide, personalized combinations	Moderate concordance, improving with combinatorial profiling	Ongoing; PDOs assist in drug repurposing and individual sensitivity assessment

In 2021, trials continued to focus on predicting drug sensitivity and response. NCT04768270 (February 2021) and NCT05175326 (January 2022) investigated the utility of ovarian cancer organoids for drug screening and evaluation of clinical consistency. Glioma-based studies, such as NCT04865315 (HiLoGlio) and NCT04868396, investigated the establishment and drug screening potential of glioblastoma stem cell organoids. Notable trials such as NCT04906733 (Cetuximab sensitivity in colon cancer) and NCT05007379 (CARMA in breast cancer) demonstrated that PDOs models could guide therapeutic decisions based on drug sensitivity.

The trend accelerated in 2022 with a focus on more complex and multi-dimensional applications. NCT05177432 and NCT05183425 evaluated the consistency between PDOs-guided and clinical responses in colorectal liver metastases and breast cancer. Trials like NCT05196334 (pancreatic cancer) and NCT05203549 (gastric cancer) further validated the clinical relevance of PDOs models in predicting chemotherapy outcomes. NCT05304741 and NCT05351983 explored the role of PDOs-based drug sensitivity in colorectal and pancreatic cancers, respectively, while NCT05384184 (BORG) assessed organoid-based therapy for colorectal cancer metastases and hepatocellular carcinoma.

In 2023, the focus expanded to include next-generation sequencing (NGS) and immunotherapy. NCT05634694 and NCT05644743 tested the predictive accuracy of PDO models for intrahepatic cholangiocarcinoma and gastrointestinal cancer. NCT05669586 assessed the role of PDOs in predicting drug resistance in non-small cell lung cancer (NSCLC). Trials such as NCT05725200 (EVIDENT) and NCT05832398 investigated the outcomes of personalized treatments in metastatic colorectal cancer and precision chemotherapy, respectively. NCT05913141 (PDO-TIL) focused on liver cancer drug screening, while NCT05955196 evaluated immune microenvironment modulation in colon cancer through CD47-SIRPα inhibitors. More recent trials in late 2023 and 2024 have sought to integrate PDOs into real-world clinical decision-making. NCT06077591 and NCT06085404 focused on validating NGS-guided and organoid-guided therapies for advanced solid tumors and ovarian cancer. NCT06102824 (ORIENTA) and NCT06155305 (ONAC) tested the efficacy of organoid-based drug sensitivity in advanced breast cancer and neoadjuvant chemotherapy. The most recent trial, NCT06195150 (ITHORinVHL), initiated in January 2024, targets Von Hippel-Lindau-related renal cancer, exploring the role of PDOs in overcoming intra- and inter-tumoral heterogeneity. Overall, these trials demonstrate a consistent and growing trend toward the clinical integration of PDOs in oncology. The versatility of organoids in drug sensitivity testing, sequencing, and disease modeling underscores their value in guiding personalized treatments and improving clinical outcomes. The increasing number of multi-center and multi-phase trials reflects a shift from experimental to more applied clinical use, positioning PDOs as pivotal tools in precision oncology. Time-to-decision is a key variable in clinical translation. On average, PDOs take 10–21 days to establish and expand to sufficient size for drug screening, with success rates varying by cancer type (60%–80%). Costs associated with growth factors, ECM matrices, and labor can be significant. However, innovations in bioprinting, synthetic scaffolds, and microfluidic platforms have reduced costs and assay volumes, improving the scalability and viability of clinical PDO pipelines.

## 7 Integration with emerging technologies

**FIGURE 10:**
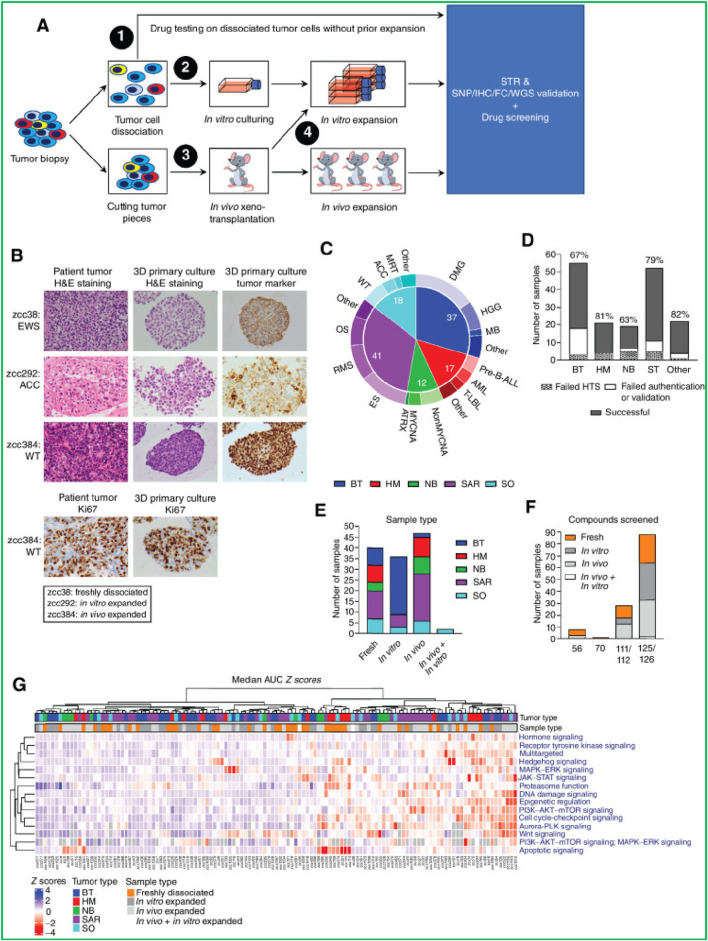
Establishment and drug screening of patient-derived tumor models. **(A)** Tumor biopsies were processed by fresh dissociation, *in vitro* culture, or *in vivo* transplantation, followed by molecular validation and drug screening. **(B)** Representative H&E and IHC staining for tumor markers and Ki-67 show similarities between patient tumors and 3D cultures in EWS (zcc38), ACC (zcc292), and WT (zcc384). **(C)** The cohort distribution of tumor types is shown. **(D)** Success and failure rates of model establishment across tumor types. **(E)** Number of samples expanded by each method. **(F)** Compounds screened per sample type. **(G)** Heatmap of drug sensitivity (median AUC Z-scores) highlights pathway-specific responses across tumor types and sample preparations.

The integration of PDOs with emerging technologies has significantly expanded their applications in cancer research and personalized medicine, enabling more comprehensive, precise, and scalable approaches to understanding and treating cancer. HTS represents one of the most impactful technological advancements in PDO research, as discussed in several examples above where HTS was employed for drug testing and therapy selection across various cancer types (Calpe and Kovacs, 2020). In addition, automated PDOs culture platforms combined with AI-driven drug response prediction are now being developed to enable real-time, patient-specific treatment recommendations in precision oncology. For instance, Using HTS technology, Fitzpatrick et al. (2017), developed a functional screening platform based on mammosphere and anoikis resistance assays to specifically identify compounds targeting cancer stem cells (CSCs) in triple-negative breast cancer. The method, validated against manual protocols, demonstrated equivalent performance in both robotic and manual formats. In this study, screening of 989 FDA-approved drugs led to the identification of three compounds capable of modulating the CSC fraction in MDA-MB-231 cells. The study clearly highlights the potential of HTS-driven strategies to uncover CSC-specific adjuvant therapies in aggressive breast cancers (Fitzpatrick et al., 2017). Moreover, by using these automated platforms, researchers can simultaneously evaluate hundreds of compounds on PDOs derived from patient tumors. For instance, a large HTS study conducted on 125 patient-derived tumor samples from children with high-risk cancers demonstrated that drug screening could identify therapeutic strategies even when genomic profiling failed to yield recommendations (Figure 10) (Mayoh et al., 2023). In 82% of cases, HTS results were available while patients were still undergoing clinical care, and the identified therapies were validated through PDX and clinical outcomes. The study also uncovered novel biomarkers of sensitivity to WEE1 and MEK inhibitors, showcasing how functional assays combined with molecular profiling can broaden therapeutic options and improve precision medicine outcomes, particularly in challenging pediatric oncology settings (Mayoh et al., 2023).

In parallel, HTS technologies are also advancing the classification of genetic variants in hereditary cancers. For example, a cDNA-based high-throughput assay was developed to functionally classify 74 BRCA1 variants of uncertain significance (VUS), particularly in the RING and BRCT domains, using BRCA1-deficient stem cells (Bouwman et al., 2013). Building on this, a large retrospective analysis involving 3,684 breast and ovarian cancer patients at Asan Medical Center demonstrated that integrating proactive HTS data enabled the reclassification of several BRCA1 VUSs into likely pathogenic or benign categories, thereby directly informing treatment strategies such as PARP inhibitor eligibility (Kim et al., 2020). These findings emphasize the growing role of functional HTS in both therapeutic optimization and genetic risk assessment within precision oncology frameworks.

**FIGURE 11:**
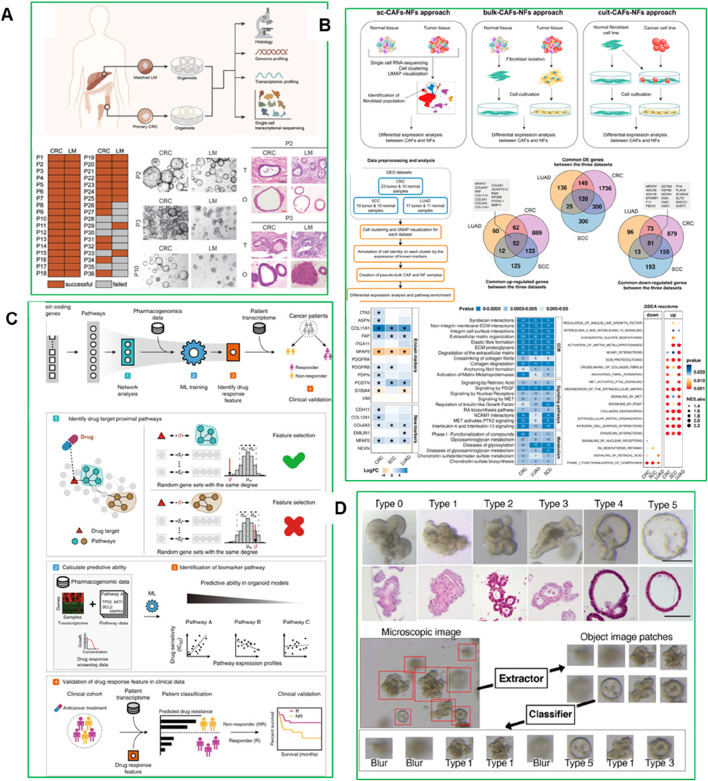
**(A)** CRLM PDO biobank established and analyzed using multiomics, showing distinct morphologies and growth patterns consistent with primary tumors (Mo et al., 2022). **(B)** Comparison of CAF and NF gene expression. scRNA-seq analysis revealed differentially expressed genes between CAFs and NFs across CRC, LUAD, and SCC. Heatmaps and enrichment analysis identified 414 CAF-specific markers and common signaling pathways linked to tumor progression (Kazakova et al., 2022). **(C)** Development of artificial intelligence (AI)-based classifier for PDOs (Okamoto et al., 2022). **(D)** Network-based ML predicts drug response in organoid models. Proximal pathways near drug targets were used to train an ML model, identifying predictive biomarkers that classified patients into responders and non-responders, validated by survival outcomes (Kong et al., 2020).

Multi-omics analysis is another critical area where PDOs are driving advancements in cancer research. Genomic sequencing of PDOs provides actionable insights into tumor-specific driver mutations and resistance pathways. Beyond genomics, proteomic and metabolomic profiling of PDOs uncovers novel therapeutic targets and metabolic vulnerabilities. These multi-dimensional datasets enable a deeper understanding of tumor biology, helping researchers identify biomarkers for therapy response and develop targeted interventions. Additionally, integration with computational tools enables the synthesis of omics data into meaningful predictions of therapeutic outcomes. For instance, Mo et al. (2022) established a biobank of 50 colorectal cancer liver metastasis (CRLM) PDOs from paired primary and metastatic tumors. In their study, Multiomics analysis, including genome, transcriptome, and single-cell sequencing, confirmed that CRLM PDOs captured intra- and interpatient heterogeneity (Figure 11A). Chemosensitivity data revealed that PDO responses to FOLFOX and FOLFIRI correlated with clinical outcomes, highlighting the predictive value of multiomics-guided PDO platforms for chemotherapy response and prognosis (Mo et al., 2022). In another study, Song et al. (2022) applied single-cell RNA sequencing (scRNA-seq) to prostate cancer biopsies and PDOs, revealing diverse epithelial cell states linked to androgen signaling and tumor progression. They identified tumor-associated club cells potentially involved in prostate carcinogenesis and showed that ERG-negative tumor cells shared characteristics with luminal epithelial cells. The study highlights the potential of single-cell analysis to uncover tumor heterogeneity and improve prostate cancer organoid models (Song et al., 2022). Similarly, Kazakova et al. (2022) performed an integrated meta-analysis of single-cell and bulk RNA sequencing data to identify CAF-specific gene expression signatures across nine cancer types. They identified 10 protein markers that showed strong positive staining in tumor stroma, offering potential biomarkers for CAF identification. The study highlights the value of transcriptome analysis using fresh tissue samples in distinguishing cancer-associated fibroblasts from normal fibroblasts, enhancing our understanding of CAF biology and their role in tumor progression (Figure 11B) (Kazakova et al., 2022). Spatial transcriptomics integrated with PDOs enables spatially resolved gene expression profiling, helping dissect tumor-immune interactions *in situ*.

Artificial intelligence (AI) and machine learning (ML) also provide powerful tools for data analysis and prediction in PDO-based studies. AI algorithms applied to large PDO datasets can predict drug responses, optimize treatment regimens, and identify novel biomarkers with unparalleled accuracy (Huang et al., 2024). These technologies also enable the automation of PDO workflows, streamlining the process of generating and analyzing organoids. For example, Kong et al. (2020) developed a machine learning framework that combines network-based analysis and pharmacogenomic data from organoid models to predict drug responses in colorectal and bladder cancer. The identified biomarkers accurately predicted the drug responses of 114 colorectal cancer patients treated with 5-fluorouracil and 77 bladder cancer patients treated with cisplatin. Concordance with independent transcriptomic and somatic mutation-based biomarkers further validated the approach, highlighting the potential of AI-driven pharmacogenomics to enhance personalized cancer therapy (Figure 11C) (Kong et al., 2020). Okamoto et al. (2022) developed an AI-based classifier to categorize CRC PDOs into six distinct morphological types based on microscopic images, revealing interpatient heterogeneity in drug response. Transcriptomic analysis showed that PDO types with high expression of ribosome biogenesis-related genes were resistant to the RNA polymerase I inhibitor CX-5461 (Figure 11D) (Okamoto et al., 2022).

## 8 Challenges and ethical considerations

So far, it is evident that PDOs offer a transformative platform for modeling patient-specific tumor biology and advancing personalized cancer therapy. However, several challenges still hinder their widespread clinical adoption and translational scalability. One major limitation is the variability in culture conditions, particularly the use of ECM-based scaffolds such as Matrigel® or BME®. These ECMs are complex, animal-derived mixtures with undefined composition and significant batch-to-batch variability, hindering standardization and regulatory compliance. Additionally, their high cost and incompatibility with high-throughput platforms pose logistical and economic barriers to widespread clinical use. However, research is already ongoing to overcome these challenges. Innovations in synthetic and defined ECM alternatives-such as peptide-based hydrogels and bioengineered scaffolds are actively being explored. A notable advancement in this area was reported by Wijnakker et al. (2025), who developed a fully defined, animal-free, integrin-targeting surface using a C-terminal fragment of Invasin, an outer membrane protein from *Yersinia*. Invasin binds to β1-integrin complexes, including α6β1, a key receptor involved in epithelial adhesion to laminin-111 (the major adhesive component of Matrigel). When Invasin was coated on standard culture plates and combined with organoid growth factors, the system supported long-term, multipassage expansion of primary epithelial cells in a 2D organoid sheet format (Wijnakker et al., 2025). Importantly, these 2D organoid sheets preserved critical features of 3D PDOs, including epithelial polarity, tight junctions, and multilineage differentiation into enterocytes, goblet cells, paneth cells, and enteroendocrine cells. The 2D configuration provided enhanced accessibility to apical and basal surfaces, facilitated imaging, and proved compatible with automated high-throughput drug screening. Moreover, the system was versatile across multiple species, including human, mouse, and even snake epithelia, highlighting its translational robustness. This innovation does not represent a return to traditional 2D cell culture but rather a re-engineered organoid platform that retains the biological complexity of PDOs while offering improved scalability, reproducibility, and clinical utility. As such, 2D organoid sheets cultured on integrin-activating surfaces represent a promising step toward defined, scalable, and cost-effective PDO systems for functional screening and translational oncology. Another study by Boretto et al. (2019) demonstrated the feasibility of generating long-term expandable PDOs from diverse endometrial pathologies, including hyperplasia, cancer, and endometriosis. These models preserved the mutational and phenotypic profiles of the original tissue and responded differentially to drug exposure, making them powerful tools for personalized therapy screening (Boretto et al., 2019). Another advancement in scalable organoid systems was reported by Shrestha et al. (2024), who developed a microarray 3D bioprinting strategy on a pillar plate platform for generating human liver organoids (HLOs) (Shrestha et al., 2024). This approach enabled high-throughput, reproducible organoid generation with minimal manual handling, addressing key limitations of traditional Matrigel dome-based cultures such as high cost and low assay throughput. By using droplet-based printing of human iPSC-derived foregut cells embedded in Matrigel onto a uniquely designed pillar plate, the team achieved uniform organoid formation with low coefficient of variation (15%–18%) and preserved cell viability. Remarkably, the organoids maintained robust functionality including albumin secretion, CYP3A4 activity, and consistent IC_50_ values for drug toxicity testing (e.g., 6.2 ± 1.6 μM for sorafenib) despite using 10–50 times less culture volume. This miniaturized, scalable platform significantly enhances the predictive power of liver toxicity assays and offers a cost-effective, automated solution for personalized drug screening applications (Shrestha et al., 2024).

Similarly, the development of a pan-cancer PDO platform involving over 1,000 patients enabled standardized, chemically defined culture systems and a neural network-based drug prediction model using label-free imaging (Boretto et al., 2019). Building on these developments, a recent large-scale study by Larsen et al. (2021) further advanced the clinical utility of PDOs by establishing a pan-cancer organoid platform encompassing over 1,000 patient-derived tumor cultures (Larsen et al., 2021). Using chemically defined minimal media tailored to each tumor type, the study achieved high genomic and transcriptomic concordance between the original tumors and their corresponding organoids, ensuring model fidelity. Crucially, the researchers introduced a neural-network-based, label-free imaging approach capable of predicting drug responses directly from brightfield microscopy. This innovation eliminated the need for fluorescent dyes or molecular labeling, thereby enabling rapid, scalable, and cost-effective high-throughput screening across diverse tumor types. Notably, over 70% of cultures successfully formed organoids, with approximately 24% reaching biobankable standards, demonstrating the feasibility of robust organoid production at scale (Larsen et al., 2021). Additionally, automated PDO bioreactors are being developed to streamline large-scale PDO cultures for clinical applications. As methodologies improve and standardized protocols are adopted, PDOs could serve as a cornerstone for translational cancer research, enabling personalized drug development, biomarker discovery, and precision oncology.

The regulatory landscape for PDO-based diagnostics remains underdeveloped. Establishing standard operating procedures (SOPs) for PDO culture and quality control, like Good Laboratory Practices (GLP), will be crucial for obtaining FDA approval and achieving widespread clinical adoption. The use of patient tissues requires transparent and informed consent processes to ensure ethical compliance. Furthermore, the genetic data generated from PDO studies raises concerns about data security and privacy. Robust frameworks must be implemented to safeguard this sensitive information and prevent misuse. Regulatory and commercialization hurdles further complicate the adoption of PDOs in clinical settings. The development of regulatory pathways for PDO-based diagnostics and therapies is still in its infancy. Establishing guidelines for quality control, clinical validation, and safety assessment will require collaboration between academia, industry, and regulatory agencies. These efforts are crucial in bridging the gap between PDO research and its application in personalized cancer care.

## 9 Future perspectives and conclusion

The next phase of PDO research is poised to address critical gaps in physiological fidelity, clinical integration, and scalability. While PDOs have already demonstrated utility in capturing patient-specific tumor biology and enabling individualized therapy selection, several frontiers demand deeper innovation. A major area of focus is the incorporation of the immune system into PDO models. Recent advances in co-culturing PDOs with autologous peripheral blood mononuclear cells (PBMCs), tumor-infiltrating lymphocytes (TILs), and dendritic cells have enabled preliminary modeling of immune checkpoint responses. However, these systems remain limited by short-term viability, immune cell exhaustion, and lack of vascular and lymphatic context. Future models must improve cytokine support, enable longer co-culture durations, and ideally incorporate vascularization or lymphoid-like niches to better emulate *in vivo* immune-tumor dynamics.

Another promising but nascent frontier is the integration of patient-specific microbiomes. Evidence increasingly links microbiota composition to therapeutic efficacy and resistance, particularly in gastrointestinal and genitourinary cancers. However, co-culturing live microbial communities with PDOs is technically challenging due to differing oxygen requirements, risks of overgrowth or contamination, and the need for anaerobic containment systems. Emerging microfluidic or gut-on-a-chip platforms may provide feasible solutions, enabling controlled and spatially defined microbial exposure within PDO environments. These systems could eventually support microbiome-based stratification or adjuvant therapy design, although translational readiness remains limited.

On the technological front, advances in ECM-substitute scaffolds (e.g., xeno-free synthetic hydrogels), microfluidic bioreactors, and robotics are gradually addressing the issues of batch-to-batch variability, manual labor intensity, and reproducibility. Standardization of culture protocols, readouts, and quality control metrics is essential to ensure reproducibility across clinical laboratories. These developments will be particularly critical for global implementation in lower-resource settings.

Finally, the widespread clinical adoption of PDO-guided therapies will depend on robust clinical validation, integration with genomic and pharmacologic data, and alignment with evolving regulatory frameworks. While several ongoing clinical trials are evaluating the predictive accuracy of PDO-based drug screening, formal regulatory endorsement will require harmonized standards for patient consent, biospecimen handling, assay reproducibility, and clinical decision interpretation. The emergence of harmonized biobank networks and real-time clinical interfaces will be pivotal in translating PDO insights into actionable therapies. Through coordinated progress across biological, technological, and regulatory domains, PDOs are poised to become an indispensable component of the precision oncology toolkit reshaping not only how we screen drugs, but how we understand, stratify, and ultimately treat cancer at the individual level.
